# Synaptic Drive onto Inhibitory and Excitatory Principal Neurons of the Mouse Lateral Superior Olive

**DOI:** 10.1523/ENEURO.0106-25.2025

**Published:** 2025-05-08

**Authors:** Hariprakash Haragopal, Mara J. Voytek, Bradley D. Winters

**Affiliations:** ^1^Department of Biological Sciences and University Hospitals – NEOMED Hearing Research Center, Northeast Ohio Medical University, Rootstown, Ohio, 44272; ^2^Brain Health Research Institute, Kent State University, Kent, Ohio, 44242

**Keywords:** auditory, brainstem, lateral superior olive, sound localization

## Abstract

Principal neurons (PNs) of the lateral superior olive (LSO) are a critical component of brain circuits that compare information between the two ears to extract sound source-location-related cues. LSO PNs are not a homogenous group but differ in their transmitter type, intrinsic membrane properties, and projection pattern to higher processing centers in the inferior colliculus. Glycinergic inhibitory LSO PNs have higher input resistance than glutamatergic excitatory LSO PNs (∼double). This suggests that the inhibitory cell type has a lower minimum input or signal intensity required to produce an output (activation threshold) which may impact how they integrate binaural inputs. However, cell-type-specific differences in the strength of synaptic drive could offset or accentuate such differences in intrinsic excitability and have not been assessed. To evaluate this possibility, we used a knock-in mouse model to examine spontaneous and electrically stimulated (evoked) synaptic events in LSO PN types using voltage-clamp technique. Both excitatory and inhibitory spontaneous postsynaptic currents were larger in inhibitory LSO PNs, but evoked events were not. Additionally, we found that LSO PN types had inputs with similar short-term plasticity and number of independent fibers. An important contrast was that inhibitory LSO PNs received inhibitory inputs with slower decay kinetics which could impact integrative functions. These data suggest that synaptic inputs onto LSO PNs are unlikely to offset excitability differences. Differences in activation threshold along with transmitter type and projection laterality may allow for distinct roles for LSO PN types in inferior colliculus information processing.

## Significance Statement

Lateral superior olive (LSO) neurons compare information between the two ears to extract location-related cues. LSO neurons differ in the transmitters they release and their projection pattern to higher processing centers, but their relative functions, synapses, and targets are not fully understood. Inhibitory LSO neurons have higher intrinsic excitability, requiring less current to drive them to fire action potentials, but it was not known whether there are cell-type-specific synaptic input differences that offset or accentuate their excitability differences. We found that the synaptic inputs to these neurons have largely similar strength, number, and short-term plasticity. This suggests that membrane excitability differences between LSO neuron types are an important factor for understanding transfer of LSO information to higher processing centers.

## Introduction

Interaural time and level differences (ITDs, ILDs) are useful for horizontal/azimuth sound localization. Circuits in the brainstem of mammals compare synaptic inputs driven by each ear to extract this information. Principal neurons (PNs) of the lateral superior olive (LSO) are distinct from olivocochlear neurons found in the same region in rodents and project via the lateral lemniscus to the inferior colliculus (IC; Sterenborg et al., 2010; Friauf et al., 2019; Williams et al., 2022; Frank et al., 2023; Maraslioglu-Sperber et al., 2024). These neurons compare excitatory inputs driven by the ipsilateral ear and inhibitory inputs driven by the contralateral ear. In this canonical circuit, ipsilateral excitation comes directly from glutamatergic spherical bushy cells in the anteroventral cochlear nucleus (AVCN) while inhibitory inputs arrive via the glycinergic relay neurons in the ipsilateral medial nucleus of the trapezoid body (MNTB) which are driven by globular bushy cells in the contralateral AVCN (for review, see Grothe et al., 2010; Friauf et al., 2019; Joris and van der Heijden, 2019; Yin et al., 2019). Subtractive analysis of these inputs gives LSO PNs intrinsic sensitivity to ITDs and ILDs (Joris and Yin, 1995; Tollin, 2005). Classically the role of LSO PNs was thought to be detection of ongoing ILDs (Tsuchitani and Boudreau, 1966; Boudreau and Tsuchitani, 1968; Tsuchitani, 1977); however, their function in detection of ITDs for the beginning of sounds (onsets), amplitude modulations, and transient broadband sounds is increasingly appreciated (Beiderbeck et al., 2018; Franken et al., 2018, 2021; Joris and Trussell, 2018; Ono et al., 2020; Chen and Song, 2024) and there may be cellular diversity to support both roles in the LSO PN population (Haragopal and Winters, 2023).

Not only does the function of LSO PNs depend on the relative weight of their inhibitory and excitatory inputs encoding a location, but the LSO PNs themselves also consist of inhibitory (glycinergic) and excitatory (glutamatergic) transmitter types (Glendenning et al., 1992; Henkel and Brunso-Bechtold, 1995; Fredrich et al., 2009; Ito and Oliver, 2010; Ito et al., 2011; Mellott et al., 2021). In mice, glycinergic cells make up 39% of the population and glutamatergic cells make up 61% (Haragopal et al., 2023).

Inhibitory and excitatory LSO PNs also have different projection patterns to higher processing centers in the IC. In C57BL6/J mice and rats, LSO outputs are fully segregated with inhibition being ipsilateral and excitation contralateral (Ito and Oliver, 2010; Haragopal et al., 2023; but see Williams and Ryugo, 2024); however, low-frequency-hearing cats and Mongolian gerbils have both contralateral and ipsilateral excitatory LSO projections (Glendenning et al., 1992; Henkel and Brunso-Bechtold, 1995; Fredrich et al., 2009; Mellott et al., 2021). Prior studies have examined synaptic inputs to the LSO, but not with respect to the different PN types and largely from a developmental perspective (Sanes and Rubel, 1988; Sanes, 1990; Wu and Kelly, 1992; Sanes, 1993; Gillespie et al., 2005; Kandler et al., 2009; Noh et al., 2010; Case and Gillespie, 2011; Pilati et al., 2016; Gjoni et al., 2018b).

LSO PNs differ in their intrinsic membrane properties; however, their synaptic inputs have not been compared. Questions remain about how the combined synaptic and intrinsic properties of these two types of LSO PNs impact sound localization and higher auditory processing. Inhibitory LSO PNs have substantially higher input resistances (∼double on average) and correspondingly lower minimum current injection needed to elicit an action potential (rheobase), suggesting they have a lower minimum input or signal intensity required to produce an output (activation threshold) than excitatory LSO PNs (Haragopal and Winters, 2023). How this relates to LSO circuit function would depend on whether there are cell-type-specific differences in synaptic drive that might offset or accentuate intrinsic excitability differences. To determine whether this is the case, we made whole-cell recordings from identified LSO PN types in voltage-clamp mode and recorded spontaneous and electrically stimulated synaptic responses using synaptic blockers to isolate glycinergic inhibitory and glutamatergic excitatory inputs.

We found several cell-type-specific differences in synaptic drive but also many similarities. Inhibitory LSO PNs exhibited larger amplitude spontaneous excitatory and inhibitory events, but not evoked events. Spontaneous event frequencies and the number of single input fibers were similar between LSO PN types. These data suggest that cell type-specific synaptic drive does not offset intrinsic membrane excitability differences between LSO PN types and that the inhibitory LSO PNs likely have lower activation thresholds within the canonical LSO circuit. We also observed slower decay kinetics in inhibitory LSO PNs which may differentially affect integrative synaptic functions. Together, these data clarify previously observed excitability differences and lay the groundwork for future modeling and in vivo studies.

## Materials and Methods

### Animals

All animal procedures were approved by the Northeast Ohio Medical University Animal Care and Use Committee in accordance with the guidelines of the United States National Institutes of Health. Mice were procured from Jackson Labs. We produced vGlut2 reporter mice by crossing a vGlut2-cre mouse line (B6J.129S6[FVB]-Slc17a6^tm2(cre)Lowl^/MwarJ; RRID: IMSR_JAX:028863) with Ai9 tdTomato reporter mice [B6.Cg-Gt(ROSA)26Sort^m9(CAG-tdTomato)Hze^/J; RRID: IMSR_JAX:007909] to obtain red fluorescent labeling of *vGlut2*-expressing cells. Animals were then bred at Northeast Ohio Medical University maintaining a 12 h light cycle with *ad libitum* food and water. Animals of both sexes were used (62 male and 44 female). At the time of electrophysiological recordings, mice were aged 26–50 d (40 ± 0.6 average). There was no difference in age between LSO PN transmitter types (vGlut2+: 40.34 ± 0.75 d, *n* = 55; vGlut2−: 39.96 ± 0.82 d, *n* = 51, *p* = 0.73, *t* test).

### Electrophysiology

Animals were transcardially perfused under isoflurane anesthesia with oxygenated, room temperature cutting solution containing the following (in mM): 135 *N*-methyl-d-glucamine (NMDG, Sigma), 1.25 KCl, 1.25 KH_2_PO_4_, 0.5 CaCl_2_, 2.5 MgCl_2_, pH 7.35 with HCl, ∼310 mmol/kg then decapitated and the brain quickly removed. The brainstem was isolated, embedded in 1% agarose, and sliced coronally 200–230 µm thick using a vibrating microtome (7000 smz2, Campden) at room temperature. Slices were transferred to a recovery solution containing the following (in mM): 110 NaCl, 2.5 KCl, 1.5 CaCl_2_, 1.5 MgCl_2_, 25 NaHCO_3_, 1.25 NaH_2_PO_4_, 12 dextrose, 5 *N*-acetyl-ʟ-cysteine, 5 Na-ascorbate, 3 Na-pyruvate, 2 thiourea, pH 7.35 with NaOH, continuously bubbled with 5% carbogen, ∼295 mmol/kg at 35°C for 30 min and then maintained at room temperature until being transferred to the recording stage. Recordings were made in oxygenated artificial cerebrospinal fluid (ACSF) containing the following (in mM): 120 NaCl, 2.5 KCl, 1.5 CaCl_2_, 1.5 MgCl_2_, 25 NaHCO_3_, 1.25 NaH_2_PO_4_, 12 dextrose, pH 7.35 with NaOH, ∼295 mmol/kg at 35 ± 0.5°C maintained using an in-line heating system.

Neurons were targeted using differential interference contrast microscopy combined with widefield fluorescence (Axioskop 2 FS Plus, 40× NA 0.8 objective, Zeiss). LSO PNs were distinguished from olivocochlear cells based on size and shape. LSO neurons that are larger, fusiform-shaped cells are highly likely (>95%) to be PNs with prominent *I*_h_ sag currents and lacking the A-current delay-to-firing response associated with olivocochlear cells (Adam et al., 1999; Sterenborg et al., 2010; Williams et al., 2022; Maraslioglu-Sperber et al., 2024).

Whole-cell patch-clamp recordings were made using Dual IPA (Sutter Instrument) or EPC-10 USB (HEKA) amplifiers with integrated digitizers in voltage-clamp mode using thick-walled borosilicate patch pipettes (2–4 MΩ, Sutter Instrument) filled with Cs-based internal containing the following (in mM): 40 CsMeSO_3_, 70 CsCl, 5 EGTA, 10 HEPES, 10 Na_2_ phosphocreatine, ∼8 sucrose, 2 Mg-ATP, 0.3 Na-GTP, 1.8 CaCl_2_, 1.5 QX-314 (HBr), 0.02 ZD 7288, 5 4-AP, 10 TEA-Cl, 0.1% (2.68 mM) biocytin, pH 7.30 with CsOH, ∼295 mmol/kg, *E*_Cl_ = −12 mV at 35°C. Data were low-pass filtered at 5 or 2.9 kHz, digitized at 20–50 kHz, and acquired to computer using PatchMaster Next (HEKA) or SutterPatch (Sutter Instrument). A calculated liquid junction potential of −6 mV was corrected. Resting membrane potential was recorded immediately after break-in. Cells were held at −70 mV during synaptic data collection.

α-amino-3-hydroxy-5-methyl-4-isoxazolepropionic acid receptor (AMPAR)-mediated glutamatergic inputs were isolated by bath-applied pharmacological blockade of glycine receptors (1 µM strychnine, Sigma), GABA_A_ receptors (2 µM gabazine, Sigma), GABA_B_ receptors (1 µM CGP55845, Sigma), and NMDA receptors (25 µM d-AP5, Alomone Labs). Glycine receptor (GlyR)-mediated inhibition was isolated by bath-applied pharmacological blockade of GABA_A_ receptors (2 µM gabazine, Sigma), GABA_B_ receptors (1 µM CGP55845, Sigma), NMDA receptors (25 µM d-AP5, Alomone Labs), and AMPA/kainite receptors (10 µM NBQX, Alomone Labs).

### Spontaneous synaptic events

Spontaneous events were analyzed from 9 to 31 1 s long raw traces acquired at 50 kHz (excitation, mean 117 ± 17 events/cell; inhibition, 415 ± 55 events/cell). Spontaneous events were detected using a custom MATLAB GUI platform. Semiautomated detection used an estimate of the noise floor obtained by subtracting a low-pass filtered version of the raw trace (fourth-order Butterworth at 1.25 kHz cutoff) from the raw trace and a manual threshold for deviation. Exclusion of spurious events, inclusion of missed events, and rewindowing of detected events as well as separation of compound events was done manually. Peaks of compound events were counted in frequency analysis, but only single events were used for amplitudes and kinetics. Amplitude was calculated from a local baseline immediately before the event. Amplitude averages provide information on the typical strength of postsynaptic responses. Cumulative frequency distributions provide more details. These distributions can reveal the presence of multiple channel subtypes if bimodal or multimodal and help characterize the variability due to quantal parameters. Kinetic parameters analyzed were 20–80% rise times, halfwidths (event width at half amplitude), and decay time constant (tau). Series resistance (Rs) was monitored with a −6 mV hyperpolarizing test pulse at the start of each sweep and cells were excluded if Rs changed by >30%.

### Evoked synaptic responses

A glass stimulating electrode (∼20 µm inner diameter, WPI) was placed either lateral to LSO near the seventh nerve to stimulate fibers of the ventral acoustic stria for excitatory synaptic inputs or medial to the LSO to stimulate fibers from the MNTB for inhibitory synaptic inputs. Stimulation pulses were delivered with a stimulus isolator (WPI A-395 or Digitimer DS4) controlled by the recording amplifier. Rs was monitored with a −6 mV hyperpolarizing test pulse at the start of each sweep and cells were excluded if Rs changed by >30%. Minimal stimulation (>12% failures) was 40–100 µs duration and 40 sweeps recorded at a sampling rate of 20–50 kHz. For paired pulses, 40–100 repetitions were recorded with a 2 s intersweep interval. Responses were filtered with a fourth-order low-pass Butterworth filter with a cutoff of 1.25 kHz (single pulse) or 1.5 kHz (paired pulse). Response kinetics were evaluated using 20–80% rise times, halfwidth, and decay time constant. Paired-pulse ratios were determined as the ratio of the second to the first pulse response amplitude. For PPRs, cells were only included if they had at least seven trials with responses to both stimuli.

For wide stimulation range experiments, stimulation durations were 100 µs and incrementally increased at a fixed step size for current amplitudes up to 10 mA or until LSO PN responses saturated. If synaptic responses did not saturate or the baseline was more negative than −200 pA, cells were discarded. Here, a more stringent criterion of Rs < 20% change was applied where Rs was measured from a hyperpolarizing voltage step of −10 mV applied at the beginning of a sweep. Raw traces were used for input count estimation. A further inclusion criterion applied was the absence of spontaneous or compound events in the response window. The average of a 10 ms window prior to the first pulse was used as baseline for estimating response amplitudes.

For a given neuron, the number of synaptic inputs were estimated from postsynaptic current (PSC) amplitude distribution fits using a Gaussian mixture model (GMM; Lee et al., 2023). The PSC amplitude distributions were modeled as having been generated by a latent mixture of normally distributed components, with each component contributing a specific mean amplitude and variance to the overall distribution. A sum of the Gaussians of each component weighted by its prior probability provided the fit. To arrive at the fit, the PSC amplitude distributions were first fitted with 1–20 components using the fitgmdist() function in MATLAB based on an expectation-maximization (EM) algorithm running on PSC amplitudes. For each component of a *k*-component Gaussian mixture, an initial PSC amplitude was chosen at random from the data as its mean, where the default initialization method or “plus” in fitgmdist() ensured that the chosen values are as far apart as possible for improved clustering, and the variance of PSC amplitudes was treated as a diagonal element of a *k*-by-*k* diagonal covariance matrix and was identical for all the *k* components. In the expectation step, posterior probability for membership of each PSC amplitude to each component was computed using these initial values, assuming a uniform prior probability, 1/*k* for *k* components. In the maximization step, the posterior probabilities were used as weights in computing component means, covariance matrix, and mixing proportions based on the maximum likelihood approach. In the ensuing expectation step, the mixing proportions served as prior probabilities and were used along with the means and covariance matrix for updating posterior probabilities. A maximum EM iteration of 1,500 ensured that the loglikelihoods of the *k*-component GMM fits were stable to within a tolerance of 1 × 10^−7^. The estimation of the loglikelihood function for the GMM fit required the determinant of the covariance matrix that had only diagonal entries to go into the denominator. The determinant must not be zero or assume near-zero values to avoid infinite or large loglikelihoods to ensure stable fits. Thus, at each EM iteration, a non-negative regularization value of 0.01 was added to the diagonal entries of the covariance matrix and this matrix was shared by all inputs. To identify the optimal input number, Bayesian information criterion (BIC) was computed from the loglikelihood of the GMM fit. The GMM fit was considered optimal among all *k*-component GMM fits, if for any *k*, ranging from 1 through 20 components, the BIC was 20 over the minimum value (Lee et al., 2023) and had a component count estimate larger than that allowed by minimum BIC. This provided a tradeoff between overfitting and underfitting of the distribution by the model. Then, to further penalize overfitting, the number of inputs were conservatively determined as the number of modes or peaks in the fits, as these were always smaller than the number of components. Modes were identified from fits plotted using MATLAB function pdf() at fixed bin intervals of 93 pA for synaptic inhibition and at fixed intervals of 36 pA for synaptic excitation. All modes were counted regardless of their prominence as this parameter is affected by the sum of the underlying Gaussian components. The chosen bin widths above were derived from the 25th percentiles for response amplitudes for minimal stimulation, estimated more conservatively by excluding those with large amplitudes >1 nA to capture contributions from single fibers. Single fiber inputs to LSO are likely to have multiple synaptic contacts (Cant, 1984; Gjoni et al., 2018a). Minimal and maximal stimulation amplitudes used for ratiometric input counts were the smallest and largest amplitudes at which peaks were detected by our algorithm, respectively.

### Statistics

Statistical comparisons were made using Prism (GraphPad) or MATLAB. The data are shown as mean ± SEM along with the data points. Significance was assessed using an alpha level of 0.05. The data points for synaptic measurements themselves were the average of across sweep measurements for each LSO PN and statistical inferences were mainly based on these average measurements. Comparisons between transmitter types were made on these data points using two-tailed, unpaired *t* test. Spearman's *r* was used to test correlation of parameters. Cumulative spontaneous event amplitude distributions were tested using the Kolmogorov–Smirnov test.

Paired-pulse stimulation was carried out at minimal stimulation intensities to better assess short-term synaptic plasticity by only activating the same synapses. At minimal intensity, events are small and the number of trials with both first and second events could be low (minimum of 7 for inclusion). A bootstrap test was performed at an alpha level of 0.05 on the paired-pulse data from individual neurons to test whether the average paired-pulse ratios (PPRs) were borne out of an interaction of the first pulse response with those of the second pulse and not simply due to variability in responses to the first pulse. For this, a test of interaction was carried out based on bootstrapped mean PPRs where the null distribution for mean PPRs were generated with first pulse alone. A single instance of bootstrap sampling had the same number of trials that went into calculation of average PPR for the neuron. This yielded 1,000 such instances from which mean PPRs were computed, and a null distribution was constructed from the 1,000 mean PPRs calculated. This gave at least seven traces from which bootstrap distributions could be created and at least 49 unique pairs with 1,000 iterations gave sufficient sampling for neurons with larger number of traces. Thus, the *p* value was the probability of seeing in the null distribution at least the mean PPR value (if > 1) or at most the mean PPR value (if < 1) computed from data.

## Results

### Excitatory inputs

**Figure 1.**
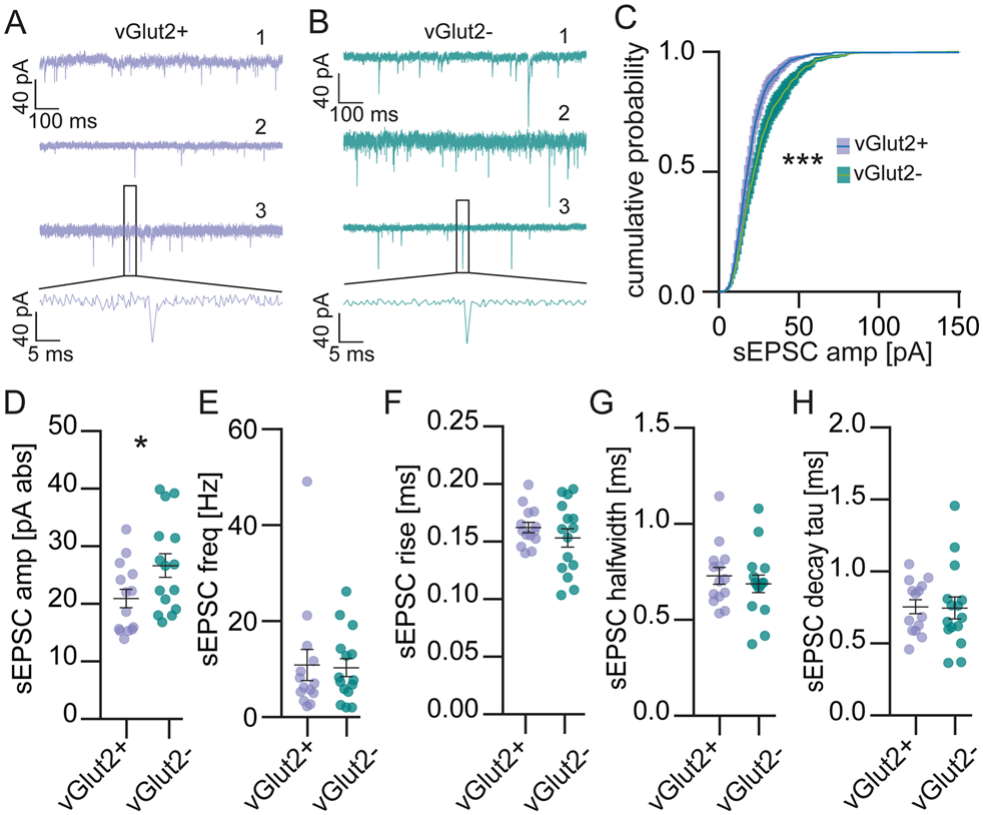
Spontaneous excitatory postsynaptic currents (sEPSCs) are larger in inhibitory LSO PNs. ***A***, Example traces from 3 vGlut2 positive (excitatory) LSO PNs. ***B***, Example traces from 3 vGlut2 negative (inhibitory) LSO PNs. ***C***, Cumulative probability of sEPSC amplitudes. ***D***, Amplitude of sEPSCs. ***E***, Frequency of sEPSCs. ***F***, sEPSC 20 to 80% rise time. ***G***, sEPSC halfwidths. ***H***, sEPSC decay tau (time constant). Cells (animals) E: *n* = 14(8), I: *n* = 15(12). Mean ± SEM. *p < 0.05, ***p < 0.001.

We used knock-in vGlut2 reporter mice to identify fluorescent excitatory LSO PNs and nonexpressing inhibitory LSO PNs by subtractive logic (Haragopal and Winters, 2023) for whole-cell voltage-clamp recordings. Since the cochlear nuclei are removed in our preparation, there is little to no action potential-driven activity in inputs to LSO PNs; therefore, spontaneous synaptic events are a good general measure of the relative number (frequency) and strength (amplitude) of synaptic inputs from all sources. Pharmacologically isolated AMPAR-mediated glutamatergic spontaneous excitatory postsynaptic currents (sEPSCs; Fig. 1*A*,*B*) exhibited larger average amplitudes in inhibitory LSO PNs [excitatory LSO PNs (E): 20.90 ± 1.60 pA, inhibitory LSO PNs (I): 26.66 ± 2.00 pA, *t* test, *p* = 0.04, Fig. 1*D*] and the cumulative probability distribution was also substantially shifted toward larger events in inhibitory LSO PNs (KS test, *D* = 0.1077, *p* < 0.001, Fig. 1*C*). The frequency of sEPSCs was similar between LSO PN types (E:10.9 ± 3.2 Hz, I:10.3 ± 1.9 Hz, *t* test, *p* = 0.90, Fig. 1*E*) as were their kinetics (rise: E: 0.160 ± 0.004 ms, I: 0.150 ± 0.008 ms, *t* test, *p* = 0.35, Fig. 1*F*; halfwidth: E: 0.730 ± 0.040 ms, I: 0.690 ± 0.046 ms, *t* test, *p* = 0.52, Fig. 1*G*; decay: E: 0.76 ± 0.05 ms, I: 0.75 ± 0.08 ms, *t* test, *p* = 0.93, Fig. 1*H*).

**Figure 2.**
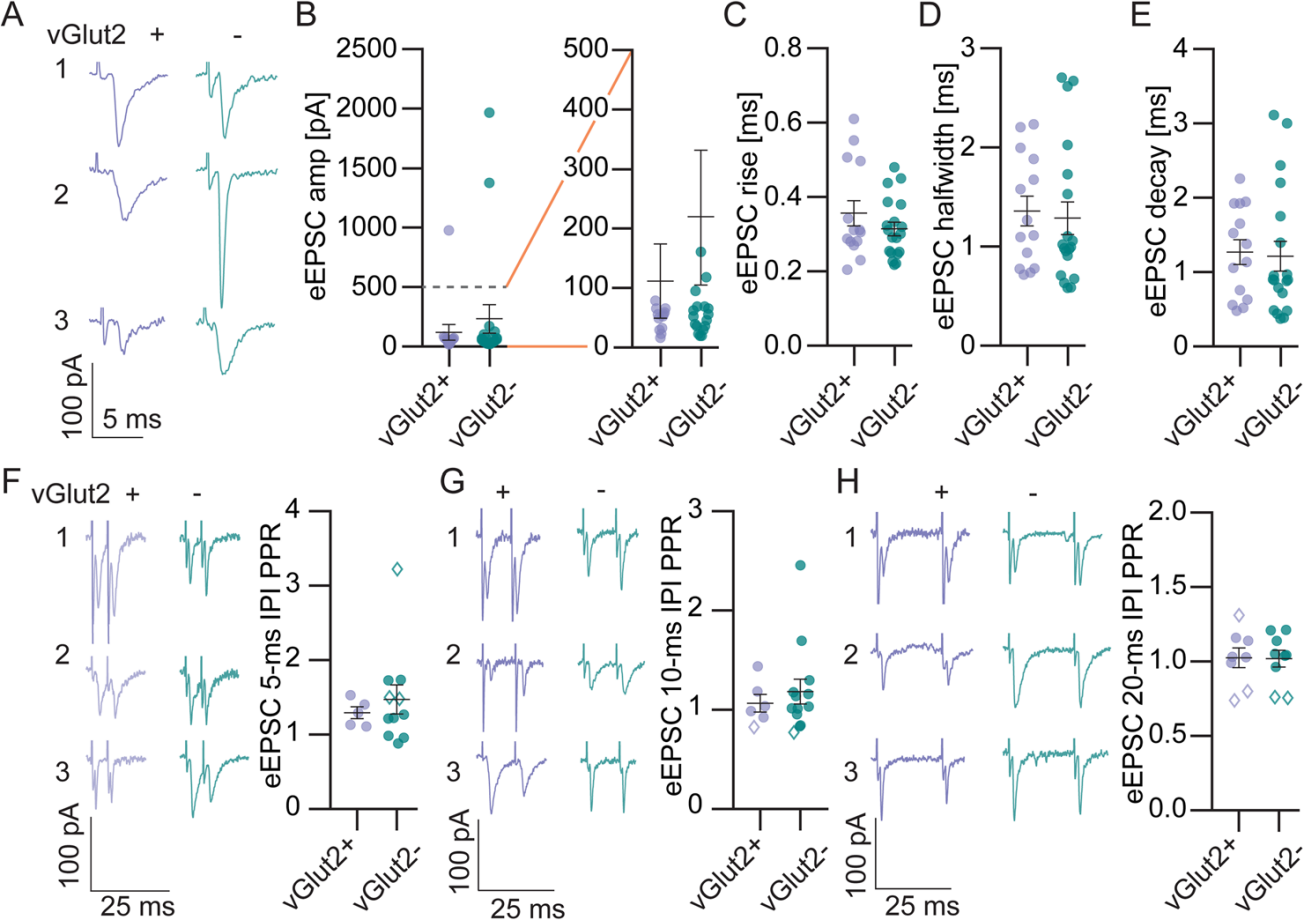
Evoked EPSCs (eEPSCs) exhibit similar amplitude, kinetics, and short-term plasticity in LSO PN types. ***A***, Example eEPSC traces at minimal stimulation from 3 vGlut2+ (left, excitatory) and 3 vGlut2− (right, inhibitory) LSO PNs. ***B***, Minimal eEPSC amplitude (all left and at expanded scale right). ***C***, Minimal eEPSC 20–80% rise time. ***D***, Minimal eEPSC halfwidth. ***E***, Minimal eEPSC decay tau. ***B–E***, Cell (animals) E: *n* = 14(10), I: *n* = 19(14). ***F***, Example eEPSC traces for a pair of stimulations at minimal stimulation intensity with 5 ms interpulse interval (IPI) from 3 vGlut2+ (left traces) and 3 vGlut2− (right traces) LSO PNs. Scale is the same for all traces. Paired-pulse ratio (right). Open diamonds in graph are cells with *p* < 0.05 in bootstrap test for individual LSO PN's mean paired-pulse ratios. E: *n* = 5(5), I: *n* = 11(10). ***G***, Same as ***F***, for 10 ms IPI. E: *n* = 6(5), I: *n* = 13(11). ***H***, 20 ms IPI. E: *n* = 8(7), I: *n* = 9(9). Mean ± SEM.

Minimal evoked EPSCs stimulated near the seventh nerve (eEPSCs, 12–90% failure rate, Fig. 2*A*) had similar average amplitude between LSO PN types (E: 120.2 ± 66.1 pA, I: 233.3 ± 118.9 pA, *t* test, *p* = 0.46, Fig. 2*B*, left and smaller events at expanded scale, *2B*, right) and included some cells with very large single fiber amplitudes in excess of 1 nA despite meeting our failure rate criteria. If we were to exclude these 3 data points, the averages would be much smaller but still not significantly different (E: 54.23 ± 5.5 pA, I: 63.90 ± 9.6, *t* test, *p* = 0.43, data not shown). We did not observe differences in minimal eEPSC kinetics between LSO PN types (rise: E: 0.36 ± 0.03 ms, I: 0.32 ± 0.02 ms, *t* test, *p* = 0.26, Fig. 2*C*; halfwidth: E: 1.36 ± 0.15 ms, I: 1.29 ± 0.17 ms, *t* test, *p* = 0.77, Fig. 2*D*; decay: E: 1.27 ± 0.17 ms, I: 1.22 ± 0.20 ms, *t* test, *p* = 0.85, Fig. 2*E*).

We examined the paired-pulse ratio of minimal eEPSCs at three interpulse intervals (IPIs, 5, 10, and 20 ms). At 5 ms IPI, the average ratios were similar between LSO PN types and slightly above 1 (E: 1.30 ± 0.07, I: 1.40 ± 0.20, *t* test, *p* = 0.56, one-sample *t* test for all PPRs shown vs PPR of 1, *p* = 0.0082, Fig. 2*F*). At larger IPIs there appeared to be little interaction between events and no differences between LSO PN types (10 ms IPI: E: 1.07 ± 0.09, I: 1.18 ± 0.13, *t* test, *p* = 0.56, one-sample *t* test for all PPRs shown vs PPR of 1, *p* = 0.13, Fig. 2*G*; 20 ms IPI: E: 1.02 ± 0.07, I: 1.02 ± 0.06, *t* test, *p* = 0.94, one-sample *t* test for all PPRs shown vs PPR of 1, *p* = 0.62, Fig. 2*H*). We used minimal stimulation for our PPR analysis to ensure interaction at the same synapses. Thus, there were a lot of trials with failures. In some cases, the number of trials in which responses to both first and second stimulation were present was relatively small (at least seven trials to be accepted). Additionally, minimal stimulation events are usually quite small amplitude raising the question of how random amplitude variability might influence PPR on a trial-by-trial basis. This might draw the computed mean PPR value away from the true mean PPR value regardless of event interaction. To address this possibility, we ran an analysis in which for each neuron the mean PPR from first and second events was compared with those from a bootstrapped set of all first events. Only a few LSO PNs exhibited significant interaction of first pulse responses on those of the second pulses (bootstrap test, *p* < 0.05, open diamonds, Fig. 2*F–H*), suggesting that concrete differences in mean PPR were unlikely at the intervals examined.

**Figure 3.**
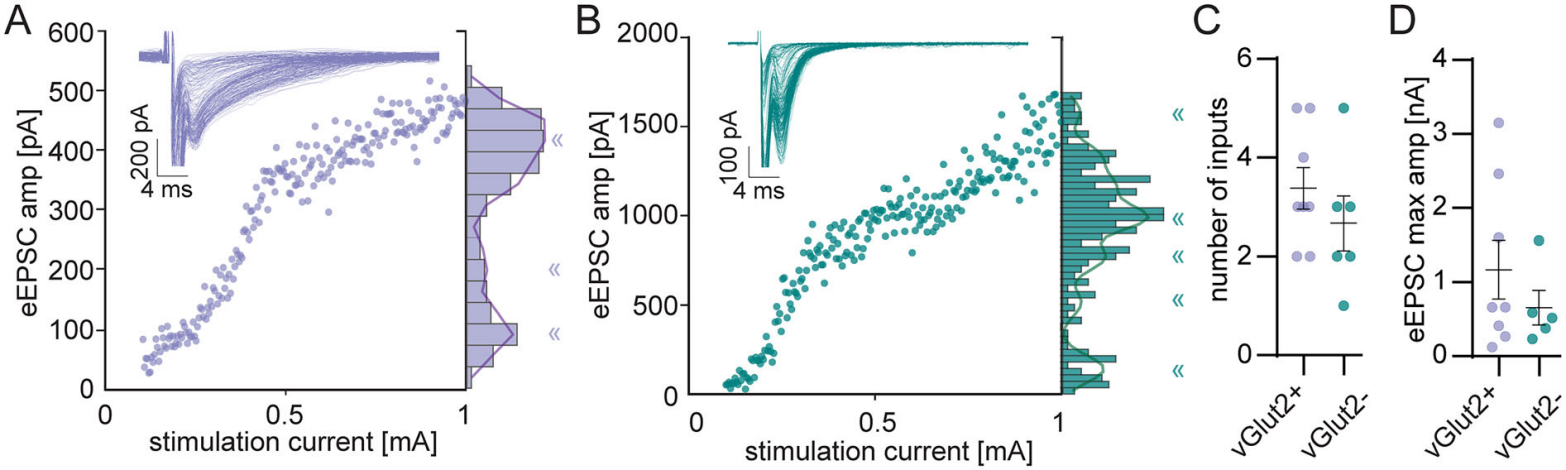
LSO PN types receive similar numbers of excitatory inputs. ***A***, Example eEPSC amplitudes plotted against stimulation intensities for an excitatory LSO PN. Overlaid response traces shown in inset. Responses saturated at 1 mA. The number of inputs for the neuron was estimated as the number of peaks in a GMM fit to the histograms binned at 36 pA (solid line overlaying histogram), « symbols indicate the local maxima of the fit. ***B***, Same as in ***A***, but for an inhibitory LSO PN. ***C***, The estimated number of inputs. E: *n* = 8(8), I: *n* = 6(6). ***D***, Maximal amplitudes, measured as the largest amplitude at which the GMM fit had a peak. One cell that had a single estimated input but <100 pA amplitude was discarded from maximal stimulation analyses. E: *n* = 7(7), I: *n* = 6(6). Mean ± SEM.

In a subset of experiments, we examined eEPSCs over a wide range of electrical stimulation levels from minimal to maximum plateau (Fig. 3*A*,*B*, insets). We analyzed the number of peaks using a GMM to assess the approximate number of input fibers (Fig. 3*A*,*B*) and found that inhibitory and excitatory LSO PNs had similar numbers (E: 3.4 ± 0.4; I: 2.7 ± 0.6, *p* = 0.32, Fig. 3*C*) and similar maximum amplitudes (E: 1,170.0 ± 395.6 pA; I: 658.8 ± 235.1 pA, *t* test, *p* = 0.36, Fig. 3*D*). We analyzed the ratio of maximal to minimal stimulation amplitudes for each neuron and found no difference between LSO PN types (E: 8.4 ± 2.2; I: 4.2 ± 1.7, *t* test, *p* = 0.17, data not shown); however, ratiometric estimates were higher than step-counting estimates (paired *t* test, *p* = 0.02). The two methods were well correlated with each other [Spearman (*r*) = 0.88, *p* < 0.0001].

### Inhibitory inputs

**Figure 4.**
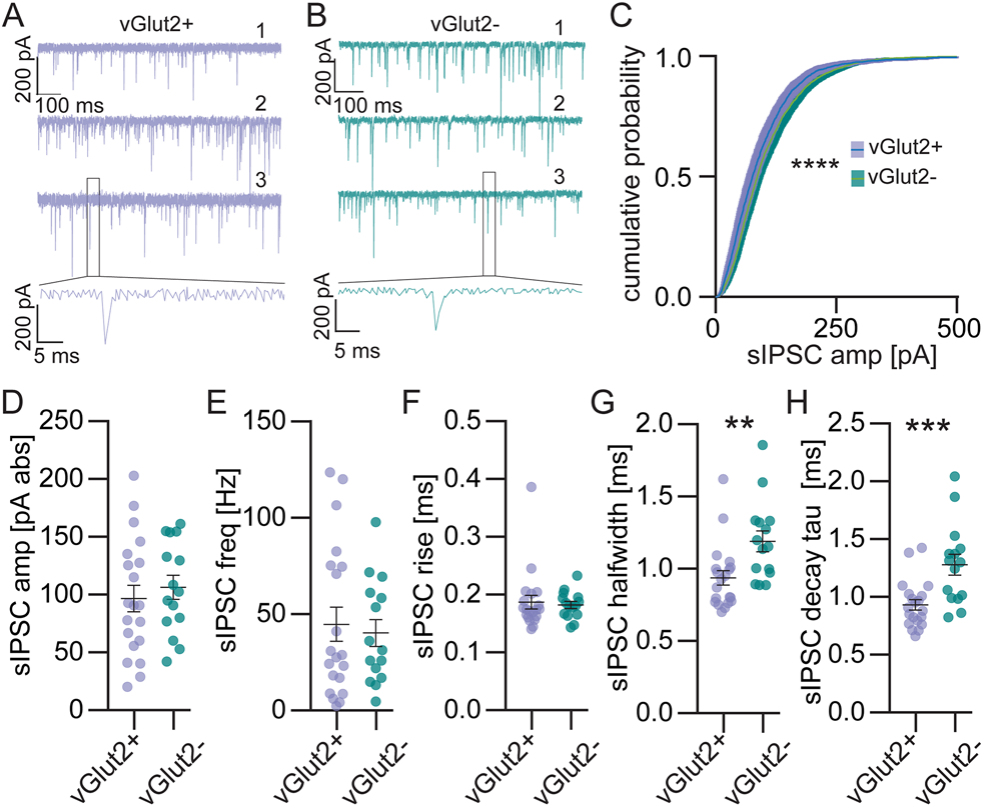
Spontaneous inhibitory postsynaptic currents (sIPSCs) have larger amplitudes and slower decay kinetics in inhibitory LSO PNs. ***A***, Example traces from 3 vGlut2 positive (excitatory) LSO PNs. High Cl^−^ internal used. ***B***, Example traces from 3 vGlut2 negative (inhibitory) LSO PNs. ***C***, Cumulative probability of sIPSC amplitudes. ***D***, Amplitude of sIPSCs. ***E***, Frequency of sIPSCs. ***F***, sIPSC 20 to 80% rise time. ***G***, sIPSC halfwidths. ***H***, sIPSC decay tau (time constant). Cells (animals) E: *n* = 20(15), I: *n* = 15(11). Mean ± SEM. **p < 0.01, ***p < 0.001, ****p < 0.0001.

Pharmacologically isolated GlyR-mediated spontaneous inhibitory postsynaptic currents (sIPSCs; Fig. 4*A*,*B*) had similar average amplitudes (E: 96.8 ± 11.4 pA, I: 106.5 ± 10.3 pA, *t* test, *p* = 0.55, Fig. 4*D*); however, the cumulative probability distribution was shifted toward more large events in inhibitory LSO PNs (KS test, *D* = 0.21, *p* < 0.0001, Fig. 4*C*), suggesting there is a population of stronger synapses that are not numerous enough to skew the average. The frequency of sIPSCs were similar between LSO PN types (E: 44.8 ± 8.9 Hz, I: 40.3 ± 6.9 Hz, *t* test, *p* = 0.71, Fig. 4*E*). Rise times for sIPSCs were similar between LSO PN types (E: 0.19 ± 0.01 ms, I: 0.18 ± 0.01 ms, *t* test, *p* = 0.74, Fig. 4*F*); however, inhibitory LSO PNs had slower sIPSC decay kinetics (halfwidth: E: 0.94 ± 0.05 ms, I: 1.20 ± 0.07 ms, *t* test, *p* = 0.0053, Fig. 4*G*; decay: E: 0.93 ± 0.05 ms, I: 1.28 ± 0.09 ms, *t* test, *p* = 0.0008, Fig. 4*H*).

**Figure 5.**
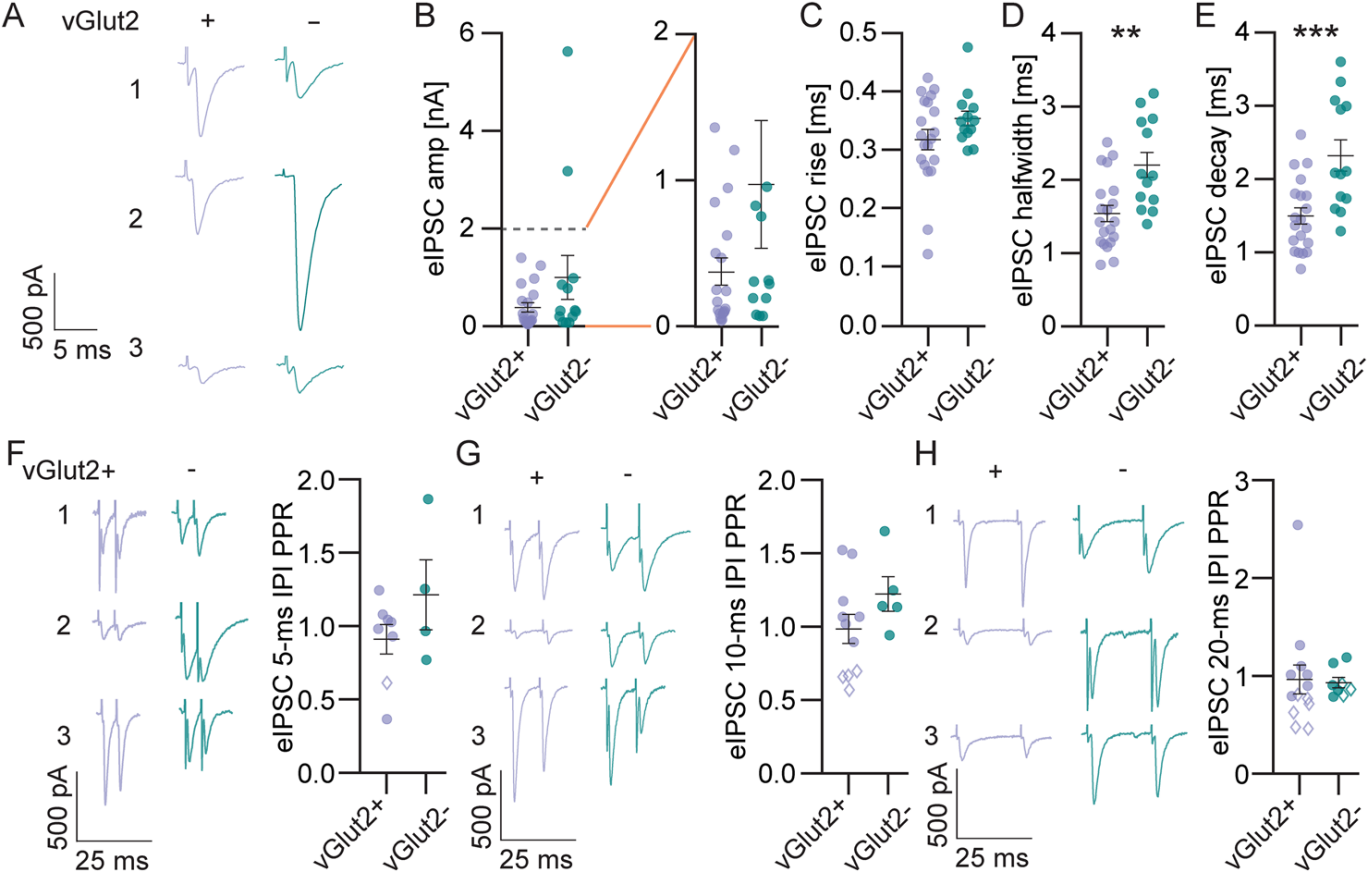
Evoked IPSCs (eIPSCs) exhibit slower decay kinetics in inhibitory LSO PNs but similar amplitudes and short-term plasticity between LSO PN types. ***A***, Example eIPSC traces at minimal stimulation from 3 vGlut2+ (left, excitatory) and 3 vGlut2− (right, inhibitory) LSO PNs. ***B***, Minimal eIPSC amplitude (all left and at expanded scale right). ***C***, Minimal eIPSC 20–80% rise time. ***D***, Minimal eIPSC halfwidth. ***E***, Minimal eIPSC decay tau. ***B–E***, Cell(animals) E: *n* = 20(15), I: *n* = 13(10). ***F***, Example eIPSC traces for a pair of stimulations at minimal stimulation intensity with 5-ms interpulse interval (IPI) from 3 vGlut2+ (left traces) and 3 vGlut2− (right traces) LSO PNs. Scale is the same for all traces. Paired-pulse ratio (right). Open diamonds in graph are cells with *p* < 0.05 in bootstrap test for individual LSO PN's mean paired-pulse ratios. E: *n* = 8(8), I: *n* = 4(4). ***G***, Same as ***F***, for 10 ms IPI. E: *n* = 11(8), I: *n* = 5(5). ***H***, 20 ms IPI. E: *n* = 13(10), I: *n* = 8(8). Mean ± SEM. **p < 0.01, ***p < 0.001.

Minimal evoked IPSCs (eIPSCs, 20–95% failure rate, Fig. 5*A*) stimulated between the MNTB and the LSO had similar average amplitude in LSO PN types (E: 389.30 ± 94.94 pA, I: 1,003.00 ± 448.80 pA, *t* test, *p* = 0.11, Fig. 5*B*, left and smaller events at expanded scale, 5*B*, right) and included two cells in the inhibitory LSO PN group with very large single fiber amplitudes in excess of 3 nA. If we were to exclude these 2 data points, the averages would be smaller, but still not significantly different (E: 389.30 ± 94.94 pA, I: 384.80 ± 99.26 pA, *t* test, *p* = 0.98, data not shown). Similar to what we saw with sIPSCs, minimal eIPSCs had similar rise times in LSO PN types (E: 0.30 ± 0.02 ms, I: 0.35 ± 0.01 ms, *t* test, *p* = 0.14, Fig. 5*C*), but slower decay kinetics in inhibitory LSO PNs (halfwidth: E: 1.54 ± 0.11 ms, I: 2.20 ± 0.17 ms, *t* test, *p* = 0.002, Fig. 5*D*; decay: E: 1.50 ± 0.11 ms, I: 2.32 ± 0.21 ms, *t* test, *p* = 0.0007, Fig. 5*E*).

We examined the paired-pulse ratio of minimal eIPSCs at three interpulse intervals as well. At all IPIs the average PPRs were similar between LSO PN types and not different from 1 (5 ms IPI: E: 0.91 ± 0.10, I: 1.20 ± 0.20, *t* test, *p* = 0.19, one-sample *t* test for all PPRs shown vs PPR of 1, *p* = 0.92, Fig. 5*F*; 10 ms IPI: E: 0.98 ± 0.10, I: 1.22 ± 0.12, *t* test, *p* = 0.17, one-sample *t* test for all PPRs shown vs PPR of 1, *p* = 0.48, Fig. 5*G*; 20 ms IPI: E: 0.96 ± 0.15, I: 0.93 ± 0.05, *t* test, *p* = 0.87, one-sample *t* test for all PPRs shown vs PPR of 1, *p* = 0.60, Fig. 5*H*). There were also few LSO PNs in which there was a significant influence of first pulse responses on those of the second pulses across IPIs (bootstrap test, *p* < 0.05, open diamonds, Fig. 5*F–H*).

**Figure 6.**
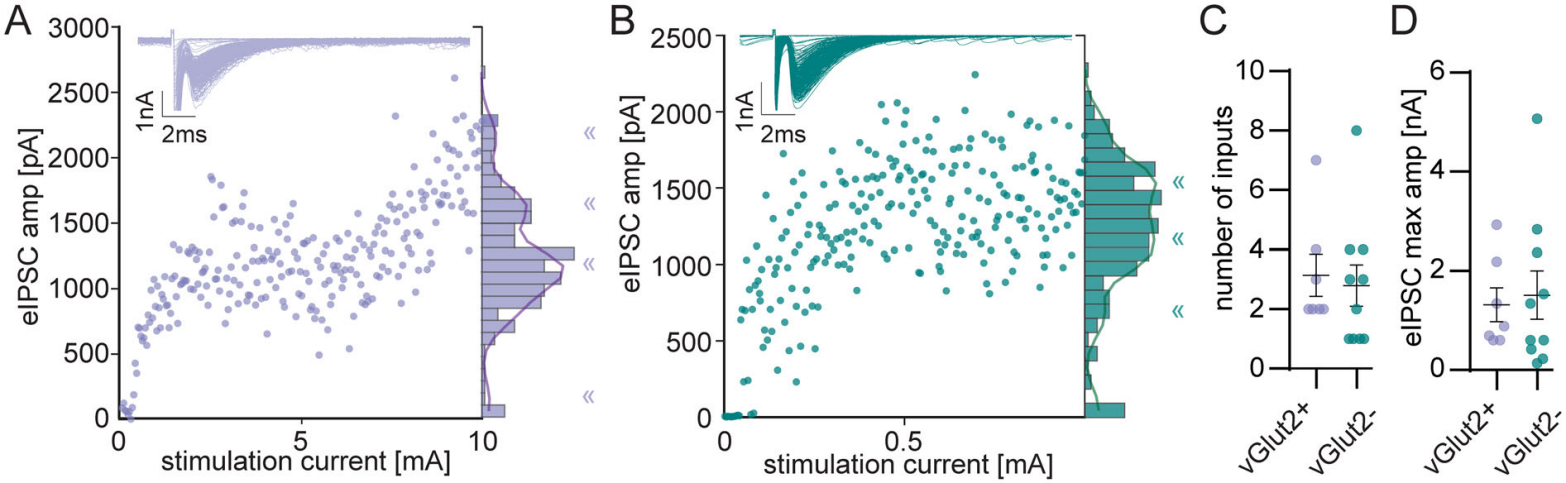
LSO PN types receive similar numbers of inhibitory inputs. ***A***, Example eIPSC amplitudes plotted against stimulation intensities for an excitatory LSO PN. Overlaid response traces shown in inset. Responses saturated at 10 mA. The number of inputs for the neuron was estimated as the number of peaks in a GMM fit to the histograms binned at 93 pA (solid line overlaying histogram), « symbols indicate the local maxima of the fit. ***B***, Same as in ***A***, but for an inhibitory LSO PN. Responses saturated at 1 mA. ***C***, The estimated number of inputs. ***D***, Maximal amplitudes, measured as the largest amplitude at which GMM fit had a peak. E: *n* = 7(6), I: *n* = 10(7). Mean ± SEM.

Similar to our observations for eEPSCs, wide stimulus range experiments with eIPSCs suggested that inhibitory and excitatory LSO PNs had similar numbers of inputs (E: 3.1 ± 0.7; I: 2.8 ± 0.7, *t* test, *p* = 0.74, Fig. 6*A–C*) and similar maximal amplitudes (E: 1,322 ± 343 pA; I: 1,516.0 ± 489.1 pA, *t* test, *p* = 0.77, Fig. 6*D*). Ratios of maximal to minimal amplitudes on a per cell basis were similar between LSO PN types as well (E: 5.6 ± 1.9; I: 3.9 ± 1.3, *t* test, *p* = 0.46, data not shown). Ratiometric estimates were similar to step-counting estimates (paired *t* test, *p* = 0.1) and the two methods were well correlated with each other [Spearman (*r*) = 0.79, *p* = 0.0003].

We estimated the whole-cell capacitance of all recorded cells in voltage-clamp mode. We found that inhibitory LSO PNs had smaller capacitance than excitatory LSO PNs (E: 17.1 ± 0.6 pF, *n* = 54; I: 14.4 ± 0.5 pF, *n* = 51; *t* test, *p* = 0.0014, data not shown).

## Discussion

Our primary objective was to determine if the strength of synaptic drive was similar between LSO PN transmitter types. We did not find any differences between LSO PN types in sEPSC or sIPSC frequency (Figs. 1*E*, 4*E*), suggesting they house similar numbers of synapses globally. We did observe that sEPSC amplitudes, both average and cumulative, were larger in inhibitory LSO PNs (Fig. 1*C*,*D*). However, we also found that the cumulative distribution of sIPSC amplitudes (Fig. 4*C*), but not the average (Fig. 4*D*), were shifted toward larger amplitudes in inhibitory LSO PNs. These data suggest the possibility that inhibitory LSO PN’s stronger excitation and stronger inhibition balance each other out, resulting in no net difference from excitatory LSO PNs. We did not observe differences in minimal eEPSC or eIPSC amplitudes (Figs. 2*B*, 5*B*) between LSO PN types. Since these would presumably be the canonical LSO circuit inputs, these data provide further support for the conclusion that the strength of synaptic drive is similar between LSO PN types. Thus, we provide strong evidence that that cell type-specific synaptic drive does not offset the higher intrinsic excitability of inhibitory LSO PNs.

Because of our electrical stimulation locations and synaptic blockers used, noncanonical LSO input sources such as ipsilateral inhibition (Weingarten et al., 2023) and contralateral excitation from nuclei of the trapezoid body (Glendenning et al., 1991) or auditory cortex (Feliciano et al., 1995; Coomes Peterson and Schofield, 2007) would not have been sampled in our evoked synaptic responses. These sources may constitute differences observed between LSO PN types in spontaneous synaptic event amplitudes. Since the function of these inputs is not understood, we cannot speculate as to what such differences might result in.

Some LSO PNs received large minimal/single fiber responses (>1 nA) despite meeting our failure rate criteria (Figs. 2*B*, 5*B*). We also observed some cells with large steps in our wide stimulus range experiments, presumably where powerful fibers were recruited above minimal stimulation intensity. The sources of these events are not known, but in the case of excitation may represent a population of cells that receive powerful inputs with multiple boutons, each of which covering multiple synapses, as has been observed in the gerbil medial superior olive (Couchman et al., 2010). Excitatory boutons with multiple synaptic components have also been described for the cat LSO at the EM level (Cant, 1984). In the case of inhibition, powerful inputs with large numbers of active zones have been described for the mouse LSO (Gjoni et al., 2018a). In either case, it is not known if the larger and smaller inputs are from different neuron populations. What we can say is that single fiber amplitudes in the LSO can have a wide range, but we did not observe differences between LSO PN types either including or excluding very large events.

While many studies have described the amplitude of synaptic inputs in LSO, methodological differences complicate direct comparison with our current study. These differences result in substantial variability and stem from the choice of recording mode (voltage-clamp vs current-clamp), developmental age, stimulation intensity, stimulation location, recording temperature, species/strain, ionic composition of external and internal solutions, synaptic blockers for isolation, and the holding potential used. Excellent recent reviews are available on the subject (Friauf et al., 2019; Yin et al., 2019) so we do not attempt to untangle this literature. One study using similar methods (Garcia-Pino et al., 2017) found that P33–35 mice exhibited glutamatergic minimal stimulation amplitudes (∼50–200 pA) that are comparable with the range of most of our eEPSCs. Likewise, another recent study (Müller et al., 2022) found minimal eIPSCs amplitudes (∼150–1,200 pA) that were similar to our observations, suggesting at least broad agreement with previous reports.

The number of independent inputs to LSO PNs is an important variable for understanding how these cells integrate synaptic drive to extract sound location-related information. Our analysis of wide stimulus range evoked amplitudes suggests that inhibitory and excitatory LSO PN types receive similar numbers of input fibers (Figs. 3*C*, 6*C*). This type of electrical stimulation assay is somewhat flawed since it is impossible to say that all fibers were stimulated/differentiated since some number may have been damaged or simply not routed through the stimulation location. Thus, the number of inputs found should be considered a lower bound used to compare between LSO PN types. We also estimated the number of inputs ratiometrically using the maximal amplitude divided by the minimal amplitude for individual neurons. These estimates were also not different between LSO PN types; however, they were larger than our step-counting estimates. This may reflect the occurrence of “multiple fiber steps” despite our small stimulation amplitude step size, thus ratiometric estimates are likely closer to the true number of inputs.

Input fiber count estimates may be affected by multiple methodological parameters such as stimulation or calculation method and there is substantial variability in prior reports. One study found the number for inhibitory inputs to LSO PNs to be ∼4 at ages P31–49 using a *K*-means clustering method for analyzing stairstep responses (Müller et al., 2019). Another prior report using ratiometric analysis of eEPSCs estimated the number of excitatory inputs to be ∼9 (Garcia-Pino et al., 2017). Ratiometric estimates using optogenetic stimulation suggested that there may be as many as 40 excitatory inputs and eight inhibitory inputs to LSO PNs (Gjoni et al., 2018b). At younger ages near hearing onset, ratiometric measurements obtained using electrical stimulation estimated ∼5 excitatory (Felix and Magnusson, 2016) and ∼10 inhibitory inputs (Hirtz et al. 2012, Clause et al. 2014).

The IPSCs onto inhibitory LSO PNs had slower decay kinetics (Figs. 4, 5). In absolute terms, the differences were small (0.26 ms wider for sIPSCs or 0.66 ms for eIPSCs), but in percent differences more substantial (24 and 35%). These kinetic differences may reflect variation in glycine receptor subunit expression and are the subject of future studies. In most neurons, if inhibitory synapses were located more distally, we would expect both the rise and decay to be affected which we did not observe. In medial superior olive neurons, there are ion channel-based mechanisms that help maintain the kinetics of propagated synaptic events (Mathews et al., 2010; Winters et al., 2017); however, whether such mechanisms are present in the LSO is unknown. Regardless, it is not known whether these width differences would substantially affect summation or integration of excitatory synaptic events, and it is a future direction to pursue computationally and experimentally.

Short-term synaptic plasticity can alter the impact of repetitive stimuli and thus could influence sound localization functions. We measured paired-pulse ratios at minimal stimulation intensities in an attempt to examine the interaction of successive stimulations on the same synapses and avoid the influence of recruitment/failure of subsets of fibers. Our results suggest that LSO PNs do not generally have paired-pulse interactions for IPIs as low as 5 ms. A few cells exhibited interactions in individual paired-pulse trials, but these cells did not consistently show interactions at the IPI tested. This was the case for both excitation and inhibition. A recent report, which also used minimal stimulation of inhibitory inputs to LSO PNs, showed that paired-pulse ratios in P31–49 mice were 0.88–0.92 at IPIs of 10 and 1,000 ms, suggesting little interaction (Müller et al., 2019). For younger mice at P12–22, another study found little paired-pulse interaction of minimally stimulated excitatory inputs to LSO PNs of ∼0.9; however, intervals longer than 20 ms were used (Felix and Magnusson, 2016). What low levels of short-term plasticity means for auditory processing is not known, but the world is rarely completely quiet, and these circuits are thought to be highly active at high rates. Being stable in the face of activity may provide benefits for information extraction. On the other hand, slices are quiet, so we do not know if in vivo short-term dynamics are different from what we observed.

We also found that the whole-cell capacitance was smaller in inhibitory LSO PNs which may contribute to their greater intrinsic membrane excitability compared with excitatory LSO PNs. These data also suggest that inhibitory LSO PNs are smaller than excitatory ones. Haragopal and Winters (2023) used *z*-stacks of fluorescence images to measure soma volumes and did not find differences between LSO PN transmitter types. However, there was a trend for inhibitory LSO PNs to have smaller somas. Additionally, capacitive measurements may encompass portions of the dendritic arbor and Haragopal and Winters did find that inhibitory LSO PNs had smaller/less complicated dendritic arbors. The capacitive measures we present here are also potentially more quantitative than the volumetric measurements because the fluorescence signal of very bright filled cell bodies tends to smear out in the *z*-axis due to the limitations of the point spread function.

Our results suggest that the greater intrinsic membrane excitability of inhibitory LSO PNs is not offset by differences in strength of synaptic drive. It is not known how having more easily excited inhibitory LSO PNs might play out in sound localization networks. Differences in activation threshold, along with transmitter type and projection laterality, may provide an additional means to segregate LSO information in higher processing centers such as the IC. It is possible that lower activation threshold would result in inhibition from LSO preceding excitation in time due to activation at smaller relative level differences. This effect could also occur from inhibitory LSO PNs having shorter spike latencies due to being driven further above their activation threshold than their excitatory counterparts. In any case, this may present a means by which inhibitory LSO PNs provide a source for “early inhibition” with shorter latency than excitation that is observed in IC neurons in vivo (Carney and Yin, 1989; Peterson et al., 2008; Voytenko and Galazyuk, 2008). The inhibitory pathway from LSO to IC is largely or entirely segregated to the ipsilateral side (Helfert et al., 1989; Saint Marie et al., 1989; Saint Marie and Baker, 1990; Glendenning et al., 1992; Henkel and Brunso-Bechtold, 1993; Ito et al., 2011; Yavuzoglu et al., 2011; Mellott et al., 2021; Haragopal et al., 2023; however, see Williams and Ryugo, 2024). Preceding either in time or relative level domain, ipsilateral inhibition may serve to sharpen the representation of auditory objects in the contralateral IC (Semple and Kitzes, 1985; Delgutte et al., 1999).

Greater understanding of the LSO→IC circuit is limited by not knowing what cell types in the IC receive inputs from LSO PN types and is an important future direction. There is however some evidence that inhibitory and excitatory LSO PNs participate in different subcircuits within the IC since ipsilateral and contralateral projections from LSO to IC target different bands or territories in cats (Shneiderman and Henkel, 1987; Loftus et al., 2004).

Although traditionally proposed to extract ILDs, LSO PNs are increasingly implicated in extraction of ITDs (for review, see Joris and Trussell, 2018; Joris and van der Heijden, 2019; Yin et al., 2019). Inhibitory LSO PNs have several intrinsic membrane properties that could provide advantages for time-coding functions. These include being more likely to present with an onset firing pattern (76%), having higher phasic spiking limit, lower maximum number of spikes, and more stable interspike intervals (Haragopal and Winters, 2023). Thus, inhibitory outputs to the ipsilateral IC from LSO may contain a preponderance of time-coding relevant information that is activated at lower sound threshold or higher sensitivity.

There is also some evidence that different types of ITD processing are segregated in the IC. ITD processing for amplitude modulations, which may be more likely to be encoded by the LSO, and fine structure ITDs which are more likely to be encoded by the medial superior olive, take place in different IC regions in gerbils (Graña et al., 2017) although it is unclear how different LSO PN cell types might be involved.

Together the work presented here suggests that inhibitory and excitatory LSO PNs have more similar synaptic inputs (with the exception of inhibitory input kinetics) than intrinsic membrane properties. Future studies will focus on specific ion channel systems involved in their intrinsic membrane differences and how differential expression may tune input/output responses for different binaural functions.

## Synthesis

Reviewing Editor: Christine Portfors, Washington State University Decisions are customarily a result of the Reviewing Editor and the peer reviewers coming together and discussing their recommendations until a consensus is reached. When revisions are invited, a fact-based synthesis statement explaining their decision and outlining what is needed to prepare a revision will be listed below. The following reviewer(s) agreed to reveal their identity: Jason Sanchez.

The reviewers agree that this is an important study investigating the synaptic differences between excitatory and inhibitory Principal Neurons (PNs) in the Lateral Superior Olive. This report shows that while excitatory and inhibitory PNs receive glutamatergic and glycinergic inputs, the synaptic properties of each cell type are broadly similar. This becomes increasingly interesting when relating it to recent work (Haragopal &Winters, 2023), which shows that inhibitory PNs are more excitable than excitatory PNs. While this work fills a significant gap in the literature, further experiments and an improved interpretation of the results are needed to raise the impact of this work.

Specific comments:

Reviewer 1:

Title: The title suggests a strong difference in the inputs to these two cell types, which is not given. The authors need to find a more suitable title.

Line 7: "activation threshold" is biophysically not well defined. Use a different terminology.

Line 10: "address this, ..." What is meant with "this", specify.

Line 12: "evoked events" do the authors refer to minimal or maximal stimulation intensity.

Line 26: "similar strength in their synaptic drive" Synaptic drive is biophysically not specified. Be more precise.

Line 27: "excitability differences" Excitability contains many different things. The authors should be more specific.

Line 35: Sentence with "via". The ending of the pathway (I guess IC) should be stated in this sentence as well.

Line 46: The reference misses the Beiderbeck 2018 citation.

Line 48: exchange block with population

Line 63: What is the difference between "lower rheobase" and "lower activation threshold"? Activation threshold is biophysically not well defined.

Line 114: The separation between LOC and LSO PN neurons as well as the population diversity of these cells needs to include the citation from Maraslioglu-Sperber 2024 throughout the manuscript.

Line 159: "first pulse response amplitudes."

Line 210: Statistics: It is required that the authors test for the distribution before they use statistical test. In some figures, the data do not appear normally distributed. In case the data is not normally distributed different statistical tests and a shift from mean to median presentation are required.

Line 235: I would recommend dividing the results in two sections with sub-headings according to the recordings of the EPSCs and the IPSCs.

Line 244: Introduce the abbreviation E and I for your two cell populations.

Line 246: "Fig. 1C) compared to VGluT2 positive LSO PNs."

Line 255: Please also give the range of number of mEPSCs you recorded per input. Did you always record and analyze the same number of mEPSCs or mIPSCs for each cell? Where the number of recorded mPSCs large enough to get distributions (e.g. >200 events)? A large event number is crucial for the mPSC analysis.

Line 280: The bin size was set 40 pA to most likely account for size of minimal fibre stimulation. But wouldn't it be more appropriate to set it to 20 pA to account for the size of mEPSCs. Repeat the analysis with a smaller bin size.

Line 283: The differences appear not significant. However, the pA size is quite different. Test if this is non-normally distributed, and if so change statistical testes.

Line 289: Explain the difference between the average mIPSC size and the cumulative data.

Line 295: Can the authors speculate, whether the different IPSC kinetics are due to different localization of synapse or possible receptor sub-units. Have the authors tried to correlate amplitude with decay?

Line 298: Have the authors recorded mPSCs in cells where they recorded ePSCs. If so, can they convert current in to vesicle number? How do they compare between different cell types and between EPSCs and IPSCs?

Line 322: How will the different cell size affect the PSC or the transformed PSP speed?

Line 326: I think the circuit discussion is detached from the real recordings and very extensive. Moreover, this section should not be the first part of the discussion, but rather the second more speculative part.

Line 372: "to pursue computationally and experimentally".

The discussion comes short on the existing literature. There have been a substantial number of reports in respect to the inputs to LSO neurons (e.g. Kandler, Gillespie,...). They should be acknowledged more.

Figure 1 and 4: A and B remove or replace "excitatory" and "inhibitory". This is irritating in respect to the PSCs that are presented. Add in all other figures the Y-labelling in an appropriate manner. E.g. in D and E: mEPSC amplitude [pA] / mIPSC or sEPSC for spontaneous if the authors don't want to term them miniature PSC. Also all units should be in brackets. This looks sloppy. Same in C for X-axis.

Figure 2 and 5: Again label the axis in a more appropriate manner, titles of the figures are unnecessary. Figure F to H. It would be better to put the data on a common X-Axis with the recorded time intervals (e.g. 0 to 25 ms). Are the data in these sub-figures obtained from the same inputs? This is important, if they originate from different inputs they are not comparable. This figure needs revision.

Reviewer 2:

Major comments:

- The significance statement, while cogent, works to undermine the new findings that you report in your manuscript. It mainly discusses the relevance of the intrinsic differences between excitatory and inhibitory PNs, and nothing about the similar synaptic properties. Maybe the authors could highlight that there are E and I LSO PNs but their functions, synapses, and downstream targets are not fully understood.

- You include 3 datapoints in eEPSC experiments (Fig 2B) and 2 datapoints in eIPSC experiments (Fig 5B) that are very large, almost an order of magnitude larger than the mean. I would personally suggest excluding them in both places, especially since including them doesn't change the significance of your tests. The excessively large amplitudes may be caused by experimental error like stimulating multiple fibers or stimulating an incorrect fiber.

- Inhibitory, GlyR synapses onto vGlut2+ and - neurons demonstrate significantly different cumulative probability and decay tau. This is one of the more interesting significant results in your manuscript. Using pharmacological blockers like CTB (Rundstrom et al., 1994) and NFA (Maleeva et al., 2017) to isolate GlyR subunits would be an essential set of experiments to add to this manuscript. This would substantiate your claim that vGlut2+ and - neurons likely express different glycine receptor subunits. Especially since this manuscript reports a lot of negative results, this would elevate the impact of your findings.

- As mentioned in the discussion, it would be interesting to test whether the longer halfwidth/decay of GlyR synapses onto inhibitory PNs would affect the summation of other PSCs with a computational model. Adding a computational model of synaptic drive to this paper would significantly enhance the impact since it would further elucidate how longer decay rates would affect LSO firing properties.

- The difference in whole-cell capacitance seems tacked on to the end of the Results. Are there any morphological differences between excitatory and inhibitory PNs that can explain this difference in capacitance? Can you fill the PNs that you patch into and image them to test for morphological differences?

- A big question is left unanswered in the Discussion. Why did you see larger sEPSC amplitudes for inhibitory PNs but not larger eEPSCs? What makes the spontaneous EPSCs different from the evoked ones? If sEPSCs are different across PN types but not eEPSCs, what does this mean about receptor expression, or signal processing?

- Overall, the Discussion is missing an impactful interpretations of the results, how they relate to findings from Haragopal &Winters (2023), and their implications on the auditory circuit. The lengthy second paragraph of the discussion does not discuss this manuscript's findings - it discusses the results from the previous manuscript (Haragopal &Winters, 2023). Perhaps before interpreting the previous report's results, you can further discuss how the combination of synaptic and intrinsic properties may contribute to auditory circuits. For example, if intrinsic properties are different but synaptic properties are similar, how would this change sound localization and overall signal processing? Even though you found some negative results, what does it all mean as a whole? While you do sprinkle some of this in throughout, the Discussion can be much more powerful.

- Your results did not show paired pulse interactions. Is this what you expected to see? You talk about it in your Discussion (386-396), however it's unclear whether these findings were surprising or not, and how it can be interpreted in the context of auditory processing.

Minor comments:

- Line 14: typo "did observed" should say "did observe".

- Lines 10-15 are somewhat jargony for an abstract.

- Lines 31-32: are ITDs and ILDs used in all vertebrates?

- Lines 33-35: further clarification on the projections of LSO PNs is needed. Do they project directly to the inferior colliculus via the lateral lemniscus, or do they synapse onto nuclei within the lateral lemniscus before projecting to the IC? The Williams et al. report seems to indicate that LSO PNs do not synapse within the lateral lemniscus. Additionally, reference to olivocochlear neurons is either not needed in this sentence, or it need to be further described as an LSO cell type that is in the efferent system. As it stands, reference to them is confusing.

- Lines 42-45: these sentences are relatively jargony and would benefit from elucidation of terms like "extraction", "onsets", etc.

- Line 49: an additional sentence highlighting the presence of excitatory and inhibitory PNs would be helpful. Not only do LSO neurons receive E:I inputs, but the cells themselves are either glutamatergic or glycinergic. The roles of these different cells in auditory circuits are mostly unknown.

- Lines 64-66: this sentence can be reworded to elevate its importance. The authors are correct that there are some very interesting questions here. Why are there excitability differences? Do these cells also exhibit different synaptic properties? How about instead, the sentence can read "While these LSO PNs differ in their intrinsic properties, their synaptic properties have not been compared. Questions remain about how the combined synaptic and intrinsic properties of these two types of principal neurons impact auditory sound localization and downstream auditory processing."

- Line 90: how many animals of each sex were used? Did you look for any sex differences?

- Line 104: please fully spell out "minutes".

- Line 170: please define PSC before using the abbreviation.

- Lines 267-269: I find this explanation to be a little confusing. Why didn't you find responses in consecutive stimulations?

- Line 280: why did you pick 40 pA to bin your responses in Figure 3A-B and Figure 6A-B?

- Lines 319-321: you add an explanation in the Discussion, but it might be helpful here to add a small interpretation. As it currently stands in the Results, the difference between the ratiometric and stepwise calculations seems like a bigger issue than it actually is.

- Line 328: this sentence is missing the work "of" between "distribution" and "sIPSC"

- Line 330: can you reiterate to the reader what you mean by the synaptic differences "balancing each other"? I assume you mean whether it balances out the intrinsic differences between excitatory and inhibitory PNs?

- Lines 337-340: the thought process is interesting, but this sentence runs on and is pretty jargony. Perhaps you can cut it up into two sentences.

- Line 350: this is a big gap in the literature: we don't know where excitatory vs. inhibitory LSO PNs project to within the IC. This is worth further highlighting as a future direction.

- Lines 361-366: again, this is a run-on sentence that is hard to follow.

- Lines 407-408: at the end of this paragraph, can you add whether you suggest the ratiometric method over the step-counting method, or vice-versa? It seems like the ratiometric method would increase your lower bound and catch any errors in the stepwise calculations, making your estimate more physiologically correct.

- Lines 416-417: at the end of this paragraph, can you add a sentence about why you think the existing literature reports a wide range of values for input fiber count? Could it be due to differences in analysis computations?

- To reduce clutter in the figures and text, consider removing halfwidth measurements (Fig 1G, 2D, 4G, 5D). Halfwidth is either not significant, or it changes as a result of a change in decay tau, which you already report.

- An interpretation of the cumulative probability distribution is missing from the manuscript. A brief description of the meaning of this metric can be added to the methods or to the Discussion when mentioned.

- Fig 1D has a p=0.04 and it looks reliant on the three possible outliers in the vGlut2- condition. How many standard deviations away from the mean are the three data points (~40 pA)? If they're more than 2 std away from the mean, consider removing them. Additionally, the bars on vGlut2- look distorted/asymmetrical.

- Fig 2B: strongly consider removing the 3 outliers and then keeping only the right panel (with y-axis from 0-500 pA).

- Fig 2F: is this result still significantly different with the removal of the outlier in vGlut2- (ratio = ~3)?

- I find Figures 3A-B and 6A-B to be somewhat unintuitive. There are multiple * symbols indicating local maxima of the model fit, but the text describes no significant difference between excitatory and inhibitor PNs for glutamatergic or glycinergic synapses. But from the figures, the difference between asterisks and local maxima visually looks like there is a significant difference.

- Fig 5B: again, strongly consider removing the two outliers and keeping the right panel only.

## Author Response

Author responses below in Blue Synthesis of Reviews:

Computational Neuroscience Model Code Accessibility Comments for Author (Required): N/A Synthesis Statement for Author (Required):

The reviewers agree that this is an important study investigating the synaptic differences between excitatory and inhibitory Principal Neurons (PNs) in the Lateral Superior Olive. This report shows that while excitatory and inhibitory PNs receive glutamatergic and glycinergic inputs, the synaptic properties of each cell type are broadly similar. This becomes increasingly interesting when relating it to recent work (Haragopal &Winters, 2023), which shows that inhibitory PNs are more excitable than excitatory PNs. While this work fills a significant gap in the literature, further experiments and an improved interpretation of the results are needed to raise the impact of this work.

We greatly appreciate the reviewer's comments and considerable effort. Both reviews were very thoughtful. There are many excellent suggestions and thought-provoking questions that led us to consider and reconsider several aspects of the work that have improved the manuscript. We have made a large number of changes to the manuscript as shown in the markup version provided. This is especially true with regard to improving interpretation of the results.

Regarding the additional experiments we must ask for some understanding. In the comments, reviewer 2 requests 2 sets of experiments and makes it clear that these experiments are not for clarification of results provided but to raise impact. To raise impact would necessarily mean that they are beyond the scope of the current study and in fact both are taken from our discussion section where we discuss future directions. We are actively following up on these findings, but neither are easy nuts to crack and will take considerable time, leading to entire publications on their own.

The information for authors at eNeuro (https://www.eneuro.org/content/general- information#types) states that additional experiments requested would be "thoroughly justified." There is no mention of a minimum impact for the journal and even negative or confirmation results are accepted (with other criteria not applicable to our report of course). Impact is subjective and not necessarily related to the quality or appropriateness of a body of experiments to answer a scientific question. Additionally, the decision on publishable unit size is a personal one and can depend on many factors such as career stage or personnel changes in a lab. We do not take lightly arguing against additional experiments and try to make a clear case below.

The reviewer requested 2 specific additional experiments. The first is to elucidate the underlying molecular differences leading to slower inhibitory postsynaptic currents in one cell type. The reviewer suggests using pharmacology to see if differential glycine receptor subunit expression might be involved, however, we are currently using another method called PatchSeq that will give us full expression information for these cell types. We will use the sequencing data to provide concrete targets to test electrophysiologically/pharmacologically, potentially using the agents suggested. In our experience, there are no simple pharmacology experiments as all agents have caveats and multiple different agents are required, making this addition non-trivial. Sequencing provides information on all potential GlyR subunits, or other factors, regardless of availability or specificity of pharmacological agents.

The second set of additional experiments requested is to use computational models to try to understand the impact of slower synaptic inhibition kinetics in one cell type on integrative sound localization functions. We are also actively working on this, but it is by no means a trivial task. We would point out that we have not previously published a computational model of this system, and no model has yet incorporated different LSO principal neuron types. The cell types need to be modeled, but there is also the need for a realistic auditory nerve/cochlear nucleus input stream that is currently lacking for mice.

We hope that the reviewers can appreciate that both of these additional sets of experiments are extensions of the current work that go well beyond the scope of our present study which was focused on finding differences in synaptic drive onto these neuron types.

Specific comments:

Reviewer 1:

Title: The title suggests a strong difference in the inputs to these two cell types, which is not given. The authors need to find a more suitable title.

The title has been modified to: Synaptic drive onto inhibitory and excitatory principal neurons of the mouse lateral superior olive Line 7: "activation threshold" is biophysically not well defined. Use a different terminology. Activation threshold has been defined in the abstract: "This suggests that the inhibitory cell type has a lower minimum input or signal intensity required to produce an output (activation threshold) which may impact how they integrate binaural inputs." What this means regarding binaural inputs/sound localization is not yet known, thus is reserved for the discussion section.

Line 10: "address this, ..." What is meant with "this", specify.

The abstract has been modified to make it more clear that the possibility outlined in the previous sentence is what is being assessed "However, cell type-specific differences in synaptic drive could offset or accentuate differences in intrinsic excitability and have not been assessed. To assess this possibility, we examined spontaneous and electrically evoked synaptic events in LSO PN cell types using voltage-clamp." Line 12: "evoked events" do the authors refer to minimal or maximal stimulation intensity.

For the sake of brevity in that abstract we left it at evoked to include all types of data provided including both minimal and maximal as well as the fine interval evoked responses.

Line 26: "similar strength in their synaptic drive" Synaptic drive is biophysically not specified. Be more precise.

The significance statement has been modified to make it more clear what is being discussed "We found that the synaptic inputs to these neurons have largely similar strength, number, and short-term plasticity." Line 27: "excitability differences" Excitability contains many different things. The authors should be more specific.

Excitability differences has been modified to "intrinsic membrane excitability differences" to make it more clear what is being discussed.

Line 35: Sentence with "via". The ending of the pathway (I guess IC) should be stated in this sentence as well.

The IC has been added as suggested.

Line 46: The reference misses the Beiderbeck 2018 citation. Beiderbeck 2018 has been added.

Line 48: exchange block with population Change made as suggested.

Line 63: What is the difference between "lower rheobase" and "lower activation threshold"? Activation threshold is biophysically not well defined.

The definition of activation threshold added to the abstract is repeated here in the introduction. Additionally, rheobase is now defined.

Line 114: The separation between LOC and LSO PN neurons as well as the population diversity of these cells needs to include the citation from Maraslioglu-Sperber 2024 throughout the manuscript.

The citation has been added as suggested.

Line 159: "first pulse response amplitudes." Modified as suggested.

Line 210: Statistics: It is required that the authors test for the distribution before they use statistical test. In some figures, the data do not appear normally distributed. In case the data is not normally distributed different statistical tests and a shift from mean to median presentation are required.

With smaller data sets, normality tests don't have much power to detect modest deviations from the Gaussian ideal. Additionally, the power of non-parametric tests is low for small data sets. Thus, we prefer to assign the type of statistic a priori based on the nature of the biological system or data type. For example, cumulative probability distributions are known to not be Gaussian and contain a larger number of values, thus we used a non-parametric test. Conversely, PSC amplitudes would be expected to vary between some small level up to some maximum, thus theoretically form a Gaussian distribution. Classic examples of bounded but considered normal distributions are things like height. We consider GraphPad a good tool and resource for this topic https://www.graphpad.com/support/faqid/1199/.

Line 235: I would recommend dividing the results in two sections with sub-headings according to the recordings of the EPSCs and the IPSCs.

Subheadings are added as suggested.

Line 244: Introduce the abbreviation E and I for your two cell populations. Acronyms have been defined as suggested.

Line 246: "Fig. 1C) compared to VGluT2 positive LSO PNs." The sentence has been modified to explicitly provide contrast between transmitter types as suggested "and the cumulative probability distribution was also substantially shifted toward larger events in inhibitory LSO PNs compared to excitatory LSO PNs (KS-test, D=0.1077, p<0.001, Fig. 1C)." Line 255: Please also give the range of number of mEPSCs you recorded per input. Did you always record and analyze the same number of mEPSCs or mIPSCs for each cell? Where the number of recorded mPSCs large enough to get distributions (e.g. >200 events)? A large event number is crucial for the mPSC analysis.

The number of analyzed events is provided in the methods section under spontaneous synaptic events "Spontaneous events were analyzed from 9 to 31 1-s long raw traces acquired at 50kHz (excitation: mean 117 {plus minus}17 events/cell; inhibition: 415 {plus minus}55 events/cell)." The smoothness of amplitude distributions and the magnitude of the error shading shown in Fig. 1C suggest that the sampling was adequate to capture the distribution.

Line 280: The bin size was set 40 pA to most likely account for size of minimal fibre stimulation. But wouldn't it be more appropriate to set it to 20 pA to account for the size of mEPSCs. Repeat the analysis with a smaller bin size.

The goal of algorithmic step counting was to avoid the bias introduced by traditional visual analysis by applying the same criteria to all cells. The bin size parameter is not necessarily a realistic representation of the biology of the system (Lee et al., 2023). Nonetheless, the rationale we provided in the methods was based on the 25th percentile of minimal stimulation amplitudes. However, these were taking into account the few large amplitude (>1nA) minimal event cells.

We have now used the 25th percentile without those events which has reduced the bin size to 36 pA for excitation. We also repeated the analysis at 20 pA and the outcome was the same with no difference between cell types but a small increase in the number of inputs (E: 3.6 {plus minus}0.3, I:

4.2 {plus minus}0.3, p = 0.2). We would prefer to keep the bin size based on a smallish evoked event, as opposed to mini/single vesicle amplitude, as this is more realistic since it is likely that each fiber covers multiple synaptic sites if not multiple boutons (Cant, 1984; Gjoni, 2017). This topic has been added to the discussion.

Line 283: The differences appear not significant. However, the pA size is quite different. Test if this is non-normally distributed, and if so change statistical testes.

See comments above on line 210.

Line 289: Explain the difference between the average mIPSC size and the cumulative data. The results section has been modified to provide some more information on the general comparison of the average vs. distribution "inhibitory postsynaptic currents (sIPSCs, Fig. 4A,B) had similar average amplitudes (E: 96.8 {plus minus}11.4 pA, I: 106.5 {plus minus}10.3 pA, t-test, p =0.55, Fig. 4D), however, the cumulative probability distribution was shifted toward more large events in inhibitory LSO PNs (KS-test, D=0.21, p<0.0001, Fig. 4C) suggesting there is a population of stronger synapses that are not numerous enough to skew the average." Line 295: Can the authors speculate, whether the different IPSC kinetics are due to different localization of synapse or possible receptor sub-units. Have the authors tried to correlate amplitude with decay? We do speculate that subunit differences are a possibility in the discussion and are actively investigating this future direction using expression profiling. The different location hypothesis is interesting. In most neurons, if inhibitory synapses were located more distally, we would expect both the rise and decay to be affected which we did not observe. In MSO neurons, there are ion-channel-based mechanisms that help maintain the kinetics of propagated synaptic events (various Golding lab papers), however, it is not known if such mechanisms are present in the LSO. This is also a future direction we are planning on looking into. This topic has been added to the discussion. Regarding correlation of amplitude with decay, we used tau for decay as it is exponential and this metric in some ways incorporates amplitude with the slope of the decay to capture the shape of events regardless of amplitude. We could run correlations on amplitude with halfwidth perhaps but this would not provide different conclusions from tau.

Line 298: Have the authors recorded mPSCs in cells where they recorded ePSCs. If so, can they convert current in to vesicle number? How do they compare between different cell types and between EPSCs and IPSCs? We do have some recordings where spontaneous and evoked data were both recorded; however, the spontaneous events would be from throughout the cell, not just the evoked pathway. We did not record asynchronous events evoked in strontium, long stimulation trains, or vary bath calcium concentrations to obtain information on vesicle number since we did not observe differences in short-term plasticity.

Line 322: How will the different cell size affect the PSC or the transformed PSP speed? Cell size/capacitance would be unlikely to affect the PSCs measured in this study with blockers etc., however, PSPs would be filtered by the membrane time constant. Haragopal 2023 found that inhibitory LSO PNs had longer membrane time constants. Thus, the much higher input resistance was not offset by smaller capacitance. In the discussion we suggest that lower capacitance may contribute to higher intrinsic excitability of the inhibitory LSO PN type as the membrane can charge somewhat faster than it might if the capacitance was the same as excitatory LSO PNs given the input resistance differences.

Line 326: I think the circuit discussion is detached from the real recordings and very extensive. Moreover, this section should not be the first part of the discussion, but rather the second more speculative part.

Our primary objective was to determine if the strength of synaptic drive was similar between LSO PN transmitter types. Thus, we lead the discussion section on this point and have substantially modified the order of paragraphs in the discussion to move the more speculative parts toward the end.

Line 372: "to pursue computationally and experimentally". The manuscript has been modified as suggested.

The discussion comes short on the existing literature. There have been a substantial number of reports in respect to the inputs to LSO neurons (e.g. Kandler, Gillespie,...). They should be acknowledged more.

We acknowledge that there have been a large number of prior studies. As we state in the discussion, excellent recent reviews are available on the subject (Friauf 2019, Yin 2019) that is complicated by a wide range of difference in experimental parameters which include the choice of recording mode (voltage-clamp vs. current-clamp), developmental age, stimulation intensity, stimulation location, recording temperature, animal age, species/strain, ionic composition of external and internal solutions, synaptic blockers for isolation, and the holding potential used. For example, the Kandler and Gillespie publications are largely developmental and focused on very young animals, but they are referred to in the introduction. Thus, we did not attempt to go over all the literature. We instead focused on several studies that were more closely matched with ours methodologically to provide some context for our results. If there are specific additional references that we have missed that are relevant we would be glad to add them to the discussion.

Figure 1 and 4: A and B remove or replace "excitatory" and "inhibitory". This is irritating in respect to the PSCs that are presented. Add in all other figures the Y-labelling in an appropriate manner. E.g. in D and E: mEPSC amplitude [pA] / mIPSC or sEPSC for spontaneous if the authors don't want to term them miniature PSC. Also all units should be in brackets. This looks sloppy. Same in C for X-axis.

All figures have been modified as suggested.

Figure 2 and 5: Again label the axis in a more appropriate manner, titles of the figures are unnecessary. Figure F to H. It would be better to put the data on a common X-Axis with the recorded time intervals (e.g. 0 to 25 ms). Are the data in these sub-figures obtained from the same inputs? This is important, if they originate from different inputs they are not comparable. This figure needs revision.

The figure axis/titles have been modified as suggested. It is not clear what is meant by a common x axis. These data are organized by interpulse interval or 5 (F), 10 (G), and 20 (H) with 3 example traces from each cell type, but not necessarily from the same cell in each panel. The scale bars have been modified to include labels on all 3 panels sets in case this was part of the issue.

Reviewer 2:

Major comments:

- The significance statement, while cogent, works to undermine the new findings that you report in your manuscript. It mainly discusses the relevance of the intrinsic differences between excitatory and inhibitory PNs, and nothing about the similar synaptic properties. Maybe the authors could highlight that there are E and I LSO PNs but their functions, synapses, and downstream targets are not fully understood.

The SS has been modified as suggested to include "These neurons occur in two types that release different transmitters and differ in their projection pattern to higher processing centers, but their functions, synapses, and targets are not fully understood." - You include 3 datapoints in eEPSC experiments (Fig 2B) and 2 datapoints in eIPSC experiments (Fig 5B) that are very large, almost an order of magnitude larger than the mean. I would personally suggest excluding them in both places, especially since including them doesn't change the significance of your tests. The excessively large amplitudes may be caused by experimental error like stimulating multiple fibers or stimulating an incorrect fiber.

We appreciate the reviewer's perspective but would prefer the larger responses to remain for several reasons. We used a quite small step size to search for individual fibers and all responses had substantial failure rates. While there are potential caveats to the electrical stimulation method, as the reviewer points out, it is commonly used. Thus, not reporting what we found might ignore something of interest to the community that is biological and might be noted in other studies. It may be the case that some cells receive large, multi-component excitatory inputs as has been described in the MSO (Couchman, 2010). Excitatory boutons with multiple synaptic components have been described for the cat LSO at the EM level (Cant, 1984). Likewise, for inhibition powerful inputs with large numbers of active zones have already been described for the mouse LSO (Gjoni, 2018). We have added a discussion of this topic to the manuscript.

- Inhibitory, GlyR synapses onto vGlut2+ and - neurons demonstrate significantly different cumulative probability and decay tau. This is one of the more interesting significant results in your manuscript. Using pharmacological blockers like CTB (Rundstrom et al., 1994) and NFA (Maleeva et al., 2017) to isolate GlyR subunits would be an essential set of experiments to add to this manuscript. This would substantiate your claim that vGlut2+ and - neurons likely express different glycine receptor subunits. Especially since this manuscript reports a lot of negative results, this would elevate the impact of your findings.

The addition of this additional research aim is discussed above with the Synthesis Statement.

- As mentioned in the discussion, it would be interesting to test whether the longer halfwidth/decay of GlyR synapses onto inhibitory PNs would affect the summation of other PSCs with a computational model. Adding a computational model of synaptic drive to this paper would significantly enhance the impact since it would further elucidate how longer decay rates would affect LSO firing properties.

The addition of this additional research aim is discussed above with the Synthesis Statement.

- The difference in whole-cell capacitance seems tacked on to the end of the Results. Are there any morphological differences between excitatory and inhibitory PNs that can explain this difference in capacitance? Can you fill the PNs that you patch into and image them to test for morphological differences? The discussion has been modified to include this topic "We also found that the whole-cell capacitance was smaller in inhibitory LSO PNs which may contribute to their greater intrinsic excitability compared to excitatory LSO PNs. Haragopal and Winters, 2023 used z-stacks of fluorescence images to measure soma volumes and did not find differences between LSO PN transmitter types. However, there was a trend for inhibitory LSO PNs to have smaller somas. Additionally, capacitive measurements may encompass portions of the dendritic arbor and Haragopal and Winters did find that inhibitory LSO PNs had smaller/less complicated dendritic arbors. The capacitive measure we present here is also potentially more quantitative than the volumetric measurements because the fluorescence signal of very bright filled cell bodies tends to smear out in the z axis due to the limitations of the point spread function.

- A big question is left unanswered in the Discussion. Why did you see larger sEPSC amplitudes for inhibitory PNs but not larger eEPSCs? What makes the spontaneous EPSCs different from the evoked ones? If sEPSCs are different across PN types but not eEPSCs, what does this mean about receptor expression, or signal processing? The discussion has been modified to include this topic "Because of our electrical stimulation locations and synaptic blockers used, non-canonical LSO input sources such as ipsilateral inhibition (Weingarten et al., 2023) and contralateral excitation from nuclei of the trapezoid body (Glendenning et al., 1991) or auditory cortex (Feliciano et al., 1995; Coomes Peterson and Schofield, 2007) would not have been sampled in our evoked synaptic responses. These sources may constitute differences observed between LSO PN types in the spontaneous synaptic event amplitude. Since the function of these inputs is not understood, we cannot speculate as to what such differences might accomplish." - Overall, the Discussion is missing an impactful interpretations of the results, how they relate to findings from Haragopal &Winters (2023), and their implications on the auditory circuit. The lengthy second paragraph of the discussion does not discuss this manuscript's findings - it discusses the results from the previous manuscript (Haragopal &Winters, 2023). Perhaps before interpreting the previous report's results, you can further discuss how the combination of synaptic and intrinsic properties may contribute to auditory circuits. For example, if intrinsic properties are different but synaptic properties are similar, how would this change sound localization and overall signal processing? Even though you found some negative results, what does it all mean as a whole? While you do sprinkle some of this in throughout, the Discussion can be much more powerful.

The discussion has been modified such that the first 2 paragraphs are current-results-centered and the subsequent paragraphs have been modified to guide readers toward the ramifications of inhibitory LSO afferents having lower activation threshold.

- Your results did not show paired pulse interactions. Is this what you expected to see? You talk about it in your Discussion (386-396), however it's unclear whether these findings were surprising or not, and how it can be interpreted in the context of auditory processing.

In some ways this was what we expected to see as the reports provided at the end of the paragraph also did not observe much short-term plasticity. As far as interpretations for auditory processing, we would say that the world is rarely completely quiet so these circuits would be highly active, sometimes at high rates. Being stable in the face of constant activity may provide benefits for information extraction. On the other hand, slices are quiet, so we do not know if in vivo short-term dynamics are different from what we observed. We have added some discussion of this to the manuscript.

Minor comments:

- Line 14: typo "did observed" should say "did observe". Corrected.

- Lines 10-15 are somewhat jargony for an abstract.

We have modified the abstract to clarify some of the jargon, stimulated for evoked and adding technique to voltage-clamp.

- Lines 31-32: are ITDs and ILDs used in all vertebrates? Interesting question and we are not sure, even fish? In any case, we do not make such a claim here but are setting the stage to discuss how it is done in those that do utilize them. We added "mammals" to the next sentence to better frame the discussion though.

- Lines 33-35: further clarification on the projections of LSO PNs is needed. Do they project directly to the inferior colliculus via the lateral lemniscus, or do they synapse onto nuclei within the lateral lemniscus before projecting to the IC? The Williams et al. report seems to indicate that LSO PNs do not synapse within the lateral lemniscus. Additionally, reference to olivocochlear neurons is either not needed in this sentence, or it need to be further described as an LSO cell type that is in the efferent system. As it stands, reference to them is confusing.

The only goal of the sentence is to define an LSO "principal neuron" as exclusive of olivocochlear neurons. Since here we provide no new information on this topic, we agree with the reviewer that it would be distracting to enter into a lengthy review of LSO efferents. To our knowledge PNs provide ascending projections that all go through the LL. There are reports of them synapsing in the LL and continuing to the IC. We have added a "for review see" reference that addresses this topic well.

- Lines 42-45: these sentences are relatively jargony and would benefit from elucidation of terms like "extraction", "onsets", etc.

Extraction has been replaced with "detection" and onsets has been replaced with "the beginning of sounds." - Line 49: an additional sentence highlighting the presence of excitatory and inhibitory PNs would be helpful. Not only do LSO neurons receive E:I inputs, but the cells themselves are either glutamatergic or glycinergic. The roles of these different cells in auditory circuits are mostly unknown.

The manuscript has been modified to highlight the introduction of I/E types as suggested.

- Lines 64-66: this sentence can be reworded to elevate its importance. The authors are correct that there are some very interesting questions here. Why are there excitability differences? Do these cells also exhibit different synaptic properties? How about instead, the sentence can read "While these LSO PNs differ in their intrinsic properties, their synaptic properties have not been compared. Questions remain about how the combined synaptic and intrinsic properties of these two types of principal neurons impact auditory sound localization and downstream auditory processing." The manuscript has been modified similar to the suggestion as the leading sentences for the paragraph.

- Line 90: how many animals of each sex were used? Did you look for any sex differences? The total number of animals used was 62 males and 44 females. This information has been added to the methods section. We have not included an analysis of sex because for some categories the number of data points would be quite low and lack statistical power.

- Line 104: please fully spell out "minutes".

The manuscript has been modified as suggested.

- Line 170: please define PSC before using the abbreviation. The manuscript has been modified as suggested.

- Lines 267-269: I find this explanation to be a little confusing. Why didn't you find responses in consecutive stimulations? In the methods section we state "Paired pulse stimulation was carried out at minimal stimulation intensities to better assess short-term synaptic plasticity by only activating the same synapses.

At minimal intensity, events are small and the number of trials with both first and second events could be low (minimum of 7 for inclusion)." This is because at minimal stimulation there are failures. The lines noted have been modified to make this more clear by adding "due to failures." - Line 280: why did you pick 40 pA to bin your responses in Figure 3A-B and Figure 6A-B? The goal of algorithmic step counting was to avoid the bias introduced by traditional visual analysis by applying the same criteria to all cells. The bin size parameter is not necessarily a realistic representation of the biology of the system (Lee et al., 2023). The rationale we provided in the methods was based on the 25th percentile of minimal stimulation amplitudes, however, these were taking into account the few large amplitude (>1nA) minimal event cells. We have now used the 25th percentile without those events which has reduced the bin size to 36 pA for excitation and 93 pA for inhibition.

- Lines 319-321: you add an explanation in the Discussion, but it might be helpful here to add a small interpretation. As it currently stands in the Results, the difference between the ratiometric and stepwise calculations seems like a bigger issue than it actually is.

We modified the manuscript to remove the "however" here and simply report the results to reduce the semblance of it being an issue.

- Line 328: this sentence is missing the work "of" between "distribution" and "sIPSC" Corrected.

- Line 330: can you reiterate to the reader what you mean by the synaptic differences "balancing each other"? I assume you mean whether it balances out the intrinsic differences between excitatory and inhibitory PNs? The sentence has been modified to make this more clear "These data suggest the possibility that inhibitory LSO PN's stronger excitation and stronger inhibition balance each other out resulting in no net difference from excitatory LSO PNs." - Lines 337-340: the thought process is interesting, but this sentence runs on and is pretty jargony. Perhaps you can cut it up into two sentences.

The sentence has been broken up and a few clarifications added "It is possible that lower activation threshold would result in inhibition from LSO preceding excitation in time due to activation at smaller relative level differences. This effect could also occur from Inhibitory LSO PNs having shorter spike latencies due to being driven further above their activation threshold than their excitatory counterparts." - Line 350: this is a big gap in the literature: we don't know where excitatory vs. inhibitory LSO PNs project to within the IC. This is worth further highlighting as a future direction.

The manuscript has been modified as suggested.

- Lines 361-366: again, this is a run-on sentence that is hard to follow.

The manuscript has been modified as suggested to break up the sentence.

- Lines 407-408: at the end of this paragraph, can you add whether you suggest the ratiometric method over the step-counting method, or vice-versa? It seems like the ratiometric method would increase your lower bound and catch any errors in the stepwise calculations, making your estimate more physiologically correct.

We agree that the step counting method is likely closer to the actual number and have noted this in the discussion "We also estimated the number of inputs ratiometrically using the maximal amplitude divided by the minimal amplitude for individual neurons. These estimates were also not different between LSO PN types; however, they were larger than our step-counting estimates. This may reflect the occurrence of "multiple fiber steps" despite our small stimulation amplitude step size, thus are likely closer to the true number of inputs." - Lines 416-417: at the end of this paragraph, can you add a sentence about why you think the existing literature reports a wide range of values for input fiber count? Could it be due to differences in analysis computations? The leading sentence to this paragraph has been modified to include some examples methodological differences "Input fiber count estimates may also be affected by multiple methodological parameters such as stimulation or calculation method and there is substantial variability in prior reports." - To reduce clutter in the figures and text, consider removing halfwidth measurements (Fig 1G, 2D, 4G, 5D). Halfwidth is either not significant, or it changes as a result of a change in decay tau, which you already report.

This is an apt point, however, halfwidth is commonly reported so some investigators expect to see it and it provides a more intuitive sense of duration. The rise and decay give further information on what is driving differences. Thus, we would prefer to report all three.

- An interpretation of the cumulative probability distribution is missing from the manuscript. A brief description of the meaning of this metric can be added to the methods or to the Discussion when mentioned.

This information has been added to the methods section as suggested "Amplitude averages provide information on the typical strength of postsynaptic responses. Cumulative frequency distributions provide more details. These distributions can reveal the presence of multiple channel sub-types if bimodal or multimodal and help characterize the variability due to quantal parameters." - Fig 1D has a p=0.04 and it looks reliant on the three possible outliers in the vGlut2- condition. How many standard deviations away from the mean are the three data points (~40 pA)? If they're more than 2 std away from the mean, consider removing them. Additionally, the bars on vGlut2- look distorted/asymmetrical.

No outliers were detected using the ROUT test in GraphPad. The vertical on the error bar had been accidentally nudged and has been corrected.

- Fig 2B: strongly consider removing the 3 outliers and then keeping only the right panel (with y- axis from 0-500 pA).

See prior response to comment 2 above.

- Fig 2F: is this result still significantly different with the removal of the outlier in vGlut2- (ratio = ~3)? A 1-sample t-test for fig 2F without highest point yields p=0.0007 so removal does not change the conclusion.

- I find Figures 3A-B and 6A-B to be somewhat unintuitive. There are multiple * symbols indicating local maxima of the model fit, but the text describes no significant difference between excitatory and inhibitor PNs for glutamatergic or glycinergic synapses. But from the figures, the difference between asterisks and local maxima visually looks like there is a significant difference.

We have changed the symbol designating local maxima to avoid confusion with significance. A and B are just examples of how cells were analyzed.

- Fig 5B: again, strongly consider removing the two outliers and keeping the right panel only. See prior response to comment 2 above.

## References

[B1] Adam TJ, Schwarz DWF, Finlayson PG (1999) Firing properties of chopper and delay neurons in the lateral superior olive of the rat. Exp Brain Res 124:489–502. 10.1007/s002210050645 10090661

[B2] Beiderbeck B, Myoga MH, Müller NIC, Callan AR, Friauf E, Grothe B, Pecka M (2018) Precisely timed inhibition facilitates action potential firing for spatial coding in the auditory brainstem. Nat Commun 9:1771. 10.1038/s41467-018-04210-y 29720589 PMC5932051

[B3] Boudreau JC, Tsuchitani C (1968) Binaural interaction in the cat superior olive S segment. J Neurophysiol 31:442–454. 10.1152/jn.1968.31.3.442 5687764

[B4] Cant NB (1984) The fine structure of the lateral superior olivary nucleus of the cat. J Comp Neurol 227:63–77. 10.1002/cne.902270108 6470211

[B5] Carney LH, Yin TC (1989) Responses of low-frequency cells in the inferior colliculus to interaural time differences of clicks: excitatory and inhibitory components. J Neurophysiol 62:144–161. 10.1152/jn.1989.62.1.144 2754468

[B6] Case DT, Gillespie DC (2011) Pre- and postsynaptic properties of glutamatergic transmission in the immature inhibitory MNTB-LSO pathway. J Neurophysiol 106:2570–2579. 10.1152/jn.00644.2010 21832038

[B7] Chen C, Song S (2024) Distinct neuron types contribute to hybrid auditory spatial coding. J Neurosci 44:e0159242024. 10.1523/JNEUROSCI.0159-24.2024 39261006 PMC11502229

[B8] Coomes PD, Schofield BR (2007) Projections from auditory cortex contact ascending pathways that originate in the superior olive and inferior colliculus. Hear Res 232:67–77. 10.1016/j.heares.2007.06.009 17643879 PMC2682707

[B9] Couchman K, Grothe B, Felmy F (2010) Medial superior olivary neurons receive surprisingly few excitatory and inhibitory inputs with balanced strength and short-term dynamics. J Neurosci 30:17111–17121. 10.1523/JNEUROSCI.1760-10.2010 21159981 PMC6634925

[B10] Delgutte B, Joris PX, Litovsky RY, Yin TC (1999) Receptive fields and binaural interactions for virtual-space stimuli in the cat inferior colliculus. J Neurophysiol 81:2833–2851. 10.1152/jn.1999.81.6.2833 10368401

[B11] Feliciano M, Saldaña E, Mugnaini E (1995) Direct projections from the rat primary auditory neocortex to nucleus sagulum, paralemniscal regions, superior olivary complex and cochlear nuclei. Aud Neurosci 1:287–308.

[B12] Felix RA, Magnusson AK (2016) Development of excitatory synaptic transmission to the superior paraolivary and lateral superior olivary nuclei optimizes differential decoding strategies. Neuroscience 334:1–12. 10.1016/j.neuroscience.2016.07.039 27476438

[B13] Frank MM, Sitko AA, Suthakar K, Torres Cadenas L, Hunt M, Yuk MC, Weisz CJC, Goodrich LV (2023) Experience-dependent flexibility in a molecularly diverse central-to-peripheral auditory feedback system. Elife 12:e83855. 10.7554/eLife.83855 36876911 PMC10147377

[B15] Franken TP, Joris PX, Smith PH (2018) Principal cells of the brainstem’s interaural sound level detector are temporal differentiators rather than integrators. Elife 7:e33854. 10.7554/eLife.33854 29901438 PMC6063729

[B14] Franken TP, Bondy BJ, Haimes DB, Goldwyn JH, Golding NL, Smith PH, Joris PX (2021) Glycinergic axonal inhibition subserves acute spatial sensitivity to sudden increases in sound intensity. Elife 10:e62183. 10.7554/eLife.62183 34121662 PMC8238506

[B16] Fredrich M, Reisch A, Illing R-B (2009) Neuronal subtype identity in the rat auditory brainstem as defined by molecular profile and axonal projection. Exp Brain Res 195:241–260. 10.1007/s00221-009-1776-7 19340418

[B17] Friauf E, Krächan EG, Müller NIC (2019) Lateral superior olive: organization, development, and plasticity. In: *The Oxford handbook of the auditory brainstem* (Kandler K, ed), pp 328–394. New York, NY: Oxford University Press.

[B18] Garcia-Pino E, Gessele N, Koch U (2017) Enhanced excitatory connectivity and disturbed sound processing in the auditory brainstem of Fragile X mice. J Neurosci 37:7403–7419. 10.1523/JNEUROSCI.2310-16.2017 28674175 PMC6596706

[B19] Gillespie DC, Kim G, Kandler K (2005) Inhibitory synapses in the developing auditory system are glutamatergic. Nat Neurosci 8:332–338. 10.1038/nn1397 15746915

[B20] Gjoni E, Aguet C, Sahlender DA, Knott G, Schneggenburger R (2018a) Ultrastructural basis of strong unitary inhibition in a binaural neuron. J Physiol 596:4969–4982. 10.1113/JP276015 30054922 PMC6187040

[B21] Gjoni E, Zenke F, Bouhours B, Schneggenburger R (2018b) Specific synaptic input strengths determine the computational properties of excitation-inhibition integration in a sound localization circuit. J Physiol 596:4945–4967. 10.1113/JP276012 30051910 PMC6187026

[B23] Glendenning KK, Masterton RB, Baker BN, Wenthold RJ (1991) Acoustic chiasm III: nature, distribution, and sources of afferents to the lateral superior olive in the cat. J Comp Neurol 310:377–400. 10.1002/cne.903100308 1723989

[B22] Glendenning KK, Baker BN, Hutson KA, Masterton RB (1992) Acoustic chiasm V: inhibition and excitation in the ipsilateral and contralateral projections of LSO. J Comp Neurol 319:100–122. 10.1002/cne.903190110 1317390

[B24] Graña GD, Hutson KA, Badea A, Pappa A, Scott W, Fitzpatrick DC (2017) The organization of frequency and binaural cues in the gerbil inferior colliculus. J Comp Neurol 525:2050–2074. 10.1002/cne.24155 27997696 PMC5473171

[B25] Grothe B, Pecka M, McAlpine D (2010) Mechanisms of sound localization in mammals. Physiol Rev 90:983–1012. 10.1152/physrev.00026.2009 20664077

[B27] Haragopal H, Winters BD (2023) Principal neuron diversity in the murine lateral superior olive supports multiple sound localization strategies and segregation of information in higher processing centers. Commun Biol 6:432. 10.1038/s42003-023-04802-5 37076594 PMC10115857

[B26] Haragopal H, Mellott JG, Dhar M, Kanel A, Mafi A, Tokar N, Winters BD (2023) Tonotopic distribution and inferior colliculus projection pattern of inhibitory and excitatory cell types in the lateral superior olive of mice. J Comp Neurol 531:1381–1388. 10.1002/cne.25515 37436768 PMC11571233

[B28] Helfert RH, Bonneau JM, Wenthold RJ, Altschuler RA (1989) GABA and glycine immunoreactivity in the Guinea pig superior olivary complex. Brain Res 501:269–286. 10.1016/0006-8993(89)90644-6 2819441

[B29] Henkel CK, Brunso-Bechtold JK (1993) Laterality of superior olive projections to the inferior colliculus in adult and developing ferret. J Comp Neurol 331:458–468. 10.1002/cne.903310403 8509504

[B30] Henkel CK, Brunso-Bechtold JK (1995) Development of glycinergic cells and puncta in nuclei of the superior olivary complex of the postnatal ferret. J Comp Neurol 354:470–480. 10.1002/cne.903540313 7608333

[B32] Ito T, Oliver DL (2010) Origins of glutamatergic terminals in the inferior colliculus identified by retrograde transport and expression of VGLUT1 and VGLUT2 genes. Front Neuroanat 4:135. 10.3389/fnana.2010.00135 21048892 PMC2967334

[B31] Ito T, Bishop DC, Oliver DL (2011) Expression of glutamate and inhibitory amino acid vesicular transporters in the rodent auditory brainstem. J Comp Neurol 519:316–340. 10.1002/cne.22521 21165977 PMC3092437

[B35] Joris PX, Yin TC (1995) Envelope coding in the lateral superior olive. I. Sensitivity to interaural time differences. J Neurophysiol 73:1043–1062. 10.1152/jn.1995.73.3.1043 7608754

[B33] Joris PX, Trussell LO (2018) The calyx of held: a hypothesis on the need for reliable timing in an intensity-difference encoder. Neuron 100:534–549. 10.1016/j.neuron.2018.10.026 30408442 PMC6263157

[B34] Joris PX, van der Heijden M (2019) Early binaural hearing: the comparison of temporal differences at the two ears. Annu Rev Neurosci 42:433–457. 10.1146/annurev-neuro-080317-061925 31018099

[B36] Kandler K, Clause A, Noh J (2009) Tonotopic reorganization of developing auditory brainstem circuits. Nat Neurosci 12:711–717. 10.1038/nn.2332 19471270 PMC2780022

[B37] Lee J, Clause A, Kandler K (2023) Structural and functional development of inhibitory connections from the medial nucleus of the trapezoid body to the superior paraolivary nucleus. J Neurosci 43:7766–7779. 10.1523/JNEUROSCI.0920-23.2023 37734946 PMC10648534

[B38] Loftus WC, Bishop DC, Saint Marie RL, Oliver DL (2004) Organization of binaural excitatory and inhibitory inputs to the inferior colliculus from the superior olive. J Comp Neurol 472:330–344. 10.1002/cne.20070 15065128

[B39] Maraslioglu-Sperber A, Pizzi E, Fisch JO, Kattler K, Ritter T, Friauf E (2024) Molecular and functional profiling of cell diversity and identity in the lateral superior olive, an auditory brainstem center with ascending and descending projections. Front Cell Neurosci 18:1354520. 10.3389/fncel.2024.1354520 38846638 PMC11153811

[B40] Mathews PJ, Jercog PE, Rinzel J, Scott LL, Golding NL (2010) Control of submillisecond synaptic timing in binaural coincidence detectors by KV1 channels. Nat Neurosci 13:601–609. 10.1038/nn.2530 20364143 PMC3375691

[B41] Mellott JG, Dhar M, Mafi A, Tokar N, Winters BD (2021) Tonotopic distribution and inferior colliculus projection pattern of inhibitory and excitatory cell types in the lateral superior olive of Mongolian gerbils. J Comp Neurol 530:506–517. 10.1002/cne.25226 34338321 PMC8716415

[B43] Müller NIC, Sonntag M, Maraslioglu A, Hirtz JJ, Friauf E (2019) Topographic map refinement and synaptic strengthening of a sound localization circuit require spontaneous peripheral activity. J Physiol 597:5469–5493. 10.1113/JP277757 31529505

[B42] Müller NIC, Paulußen I, Hofmann LN, Fisch JO, Singh A, Friauf E (2022) Development of synaptic fidelity and action potential robustness at an inhibitory sound localization circuit: effects of otoferlin-related deafness. J Physiol 600:2461–2497. 10.1113/JP280403 35439328

[B44] Noh J, Seal RP, Garver JA, Edwards RH, Kandler K (2010) Glutamate co-release at GABA/glycinergic synapses is crucial for the refinement of an inhibitory map. Nat Neurosci 13:232–238. 10.1038/nn.2478 20081852 PMC2832847

[B45] Ono M, Bishop DC, Oliver DL (2020) Neuronal sensitivity to the interaural time difference of the sound envelope in the mouse inferior colliculus. Hear Res 385:107844. 10.1016/j.heares.2019.107844 31759235 PMC6933070

[B46] Peterson DC, Voytenko S, Gans D, Galazyuk A, Wenstrup J (2008) Intracellular recordings from combination-sensitive neurons in the inferior colliculus. J Neurophysiol 100:629–645. 10.1152/jn.90390.2008 18497365 PMC2525731

[B47] Pilati N, Linley DM, Selvaskandan H, Uchitel O, Hennig MH, Kopp-Scheinpflug C, Forsythe ID (2016) Acoustic trauma slows AMPA receptor-mediated EPSCs in the auditory brainstem, reducing GluA4 subunit expression as a mechanism to rescue binaural function. J Physiol 594:3683–3703. 10.1113/JP271929 27104476 PMC4929335

[B48] Saint Marie RL, Baker RA (1990) Neurotransmitter-specific uptake and retrograde transport of [3H]glycine from the inferior colliculus by ipsilateral projections of the superior olivary complex and nuclei of the lateral lemniscus. Brain Res 524:244–253. 10.1016/0006-8993(90)90698-B 1705464

[B49] Saint Marie RL, Ostapoff EM, Morest DK, Wenthold RJ (1989) Glycine-immunoreactive projection of the cat lateral superior olive: possible role in midbrain ear dominance. J Comp Neurol 279:382–396. 10.1002/cne.902790305 2918077

[B50] Sanes DH (1990) An in vitro analysis of sound localization mechanisms in the gerbil lateral superior olive. J Neurosci 10:3494–3506. 10.1523/JNEUROSCI.10-11-03494.1990 2172478 PMC6570104

[B51] Sanes DH (1993) The development of synaptic function and integration in the central auditory system. J Neurosci 13:2627–2637. 10.1523/JNEUROSCI.13-06-02627.1993 8501528 PMC6576502

[B52] Sanes DH, Rubel EW (1988) The ontogeny of inhibition and excitation in the gerbil lateral superior olive. J Neurosci 8:682–700. 10.1523/JNEUROSCI.08-02-00682.1988 3339433 PMC6569284

[B53] Semple MN, Kitzes LM (1985) Single-unit responses in the inferior colliculus: different consequences of contralateral and ipsilateral auditory stimulation. J Neurophysiol 53:1467–1482. 10.1152/jn.1985.53.6.1467 4009228

[B54] Shneiderman A, Henkel CK (1987) Banding of lateral superior olivary nucleus afferents in the inferior colliculus: a possible substrate for sensory integration. J Comp Neurol 266:519–534. 10.1002/cne.902660406 2449472

[B55] Sterenborg JC, Pilati N, Sheridan CJ, Uchitel OD, Forsythe ID, Barnes-Davies M (2010) Lateral olivocochlear (LOC) neurons of the mouse LSO receive excitatory and inhibitory synaptic inputs with slower kinetics than LSO principal neurons. Hear Res 270:119–126. 10.1016/j.heares.2010.08.013 20813177

[B56] Tollin DJ (2005) Interaural phase and level difference sensitivity in Low-frequency neurons in the lateral superior olive. J Neurosci 25:10648–10657. 10.1523/JNEUROSCI.1609-05.2005 16291937 PMC1449742

[B57] Tsuchitani C (1977) Functional organization of lateral cell groups of cat superior olivary complex. J Neurophysiol 40:296–318. 10.1152/jn.1977.40.2.296 845625

[B58] Tsuchitani C, Boudreau JC (1966) Single unit analysis of cat superior olive S segment with tonal stimuli. J Neurophysiol 29:684–697. 10.1152/jn.1966.29.4.684 5966430

[B59] Voytenko SV, Galazyuk AV (2008) Timing of sound-evoked potentials and spike responses in the inferior colliculus of awake bats. Neuroscience 155:923–936. 10.1016/j.neuroscience.2008.06.031 18621102 PMC2577224

[B60] Weingarten DJ, Sebastian E, Winkelhoff J, Patschull-Keiner N, Fischer AU, Wadle SL, Friauf E, Hirtz JJ (2023) An inhibitory glycinergic projection from the cochlear nucleus to the lateral superior olive. Front Neural Circuits 17:1307283. 10.3389/fncir.2023.1307283 38107610 PMC10722231

[B62] Williams IR, Ryugo DK (2024) Bilateral and symmetric glycinergic and glutamatergic projections from the LSO to the IC in the CBA/CaH mouse. Front Neural Circuits 18:1430598. 10.3389/fncir.2024.1430598 39184455 PMC11341401

[B61] Williams IR, Filimontseva A, Connelly CJ, Ryugo DK (2022) The lateral superior olive in the mouse: two systems of projecting neurons. Front Neural Circuits 16:1038500. 10.3389/fncir.2022.1038500 36338332 PMC9630946

[B63] Winters BD, Jin S-X, Ledford KR, Golding NL (2017) Amplitude normalization of dendritic EPSPs at the soma of binaural coincidence detector neurons of the medial superior olive. J Neurosci 37:3138–3149. 10.1523/JNEUROSCI.3110-16.2017 28213442 PMC5373109

[B64] Wu S, Kelly J (1992) Synaptic pharmacology of the superior olivary complex studied in mouse brain slice. J Neurosci 12:3084–3097. 10.1523/JNEUROSCI.12-08-03084.1992 1494947 PMC6575641

[B65] Yavuzoglu A, Schofield BR, Wenstrup JJ (2011) Circuitry underlying spectrotemporal integration in the auditory midbrain. J Neurosci 31:14424–14435. 10.1523/JNEUROSCI.3529-11.2011 21976527 PMC3226782

[B66] Yin TCT, Smith PH, Joris PX (2019) Neural mechanisms of binaural processing in the auditory brainstem. Compr Physiol 9:1503–1575. 10.1002/cphy.c180036 31688966

